# Crystallographic, kinetic, and calorimetric investigation of PKA interactions with L-type calcium channels and Rad GTPase

**DOI:** 10.1016/j.jbc.2024.108039

**Published:** 2024-11-29

**Authors:** Randy Yoo, Omid Haji-Ghassemi, Marvin Bader, Jiaming Xu, Ciaran McFarlane, Filip Van Petegem

**Affiliations:** Department of Biochemistry and Molecular Biology, University of British Columbia, Life Sciences Institute, Vancouver, British Columbia, Canada

**Keywords:** cAMP-depending protein kinase, voltage-gated calcium channel, GTP binding protein, diabetes, synaptic plasticity, cardiac muscle, beta-adrenergic signaling, enzyme kinetics, isothermal titration calorimetry, X-ray crystallography

## Abstract

β-Adrenergic signaling activates cAMP-dependent PKA, which regulates the activity of L-type voltage-gated calcium channels such as Ca_V_1.2. Several PKA target sites in the C-terminal tail of Ca_V_1.2 have been identified, and their phosphorylation has been suggested to increase currents in specific tissues or heterologous expression systems. However, augmentation of Ca_V_1.2 currents in the heart is instead mediated by phosphorylation of Rad, a small GTPase that can inhibit Ca_V_1.2. It is unclear how each of the proposed target sites in Ca_V_1.2 and Rad rank toward their recognition by PKA, which could reveal a preferential phosphorylation. Here, we used quantitative assays on three Ca_V_1.2 and four Rad sites. Isothermal titration calorimetry and enzyme kinetics show that there are two Tiers of targets, with Ca_V_1.2 residue Ser1981 and Rad residues Ser25 and Ser272 forming tier one substrates for PKA. These share a common feature with two Arginine residues at specific positions that can anchor the peptide into the substrate binding cleft of PKA. In contrast, PKA shows minimal activity for the other, tier two substrates, characterized by low k_cat_ values and undetectable binding *via* isothermal titration calorimetry. The existence of two tiers suggests that PKA regulation of the Ca_V_1.2 complex may occur in a graded fashion. We report crystal structures of the PKA catalytic subunit with and without a Ca_V_1.2 and test the importance of several anchoring residues *via* mutagenesis. Different target sites utilize different anchors, highlighting the plasticity of PKAc to recognize substrates.

The voltage-gated Ca^2+^ channel Ca_V_1.2 is expressed in multiple tissues, including vascular smooth muscle, neurons, cardiomyocytes, and pancreatic β cells (1). Upon depolarization of the plasma membrane, opening of Ca_V_1.2 leads to a temporary influx of Ca^2+^ into the cytosol, which can trigger multiple Ca^2+^-dependent processes, including hormone release, initiating cardiac muscle contraction, and excitation-transcription coupling. Ca_V_1.2 is a multisubunit protein with a pore-forming α_1c_, an extracellular α_2_δ and a cytoplasmic β subunit. The α_1c_ subunit consists of four repeats (I–IV) containing six transmembrane helices each (Fig. 1*A*). The loop connecting repeats I and II (‘I-II loop’) forms a high-affinity binding site for the Ca_V_β subunit (2, 3, 4). Except for a small EF-hand domain, the extensive C-terminal tail of the channel has remained invisible in cryo-EM reconstructions of Ca_V_1.2 (5, 6). This tail contains well-characterized docking sites for regulatory proteins such as Calmodulin (7, 8), which binds to an IQ domain, and Junctophilin (9) (Fig. 1*A*).

**Figure 1:**
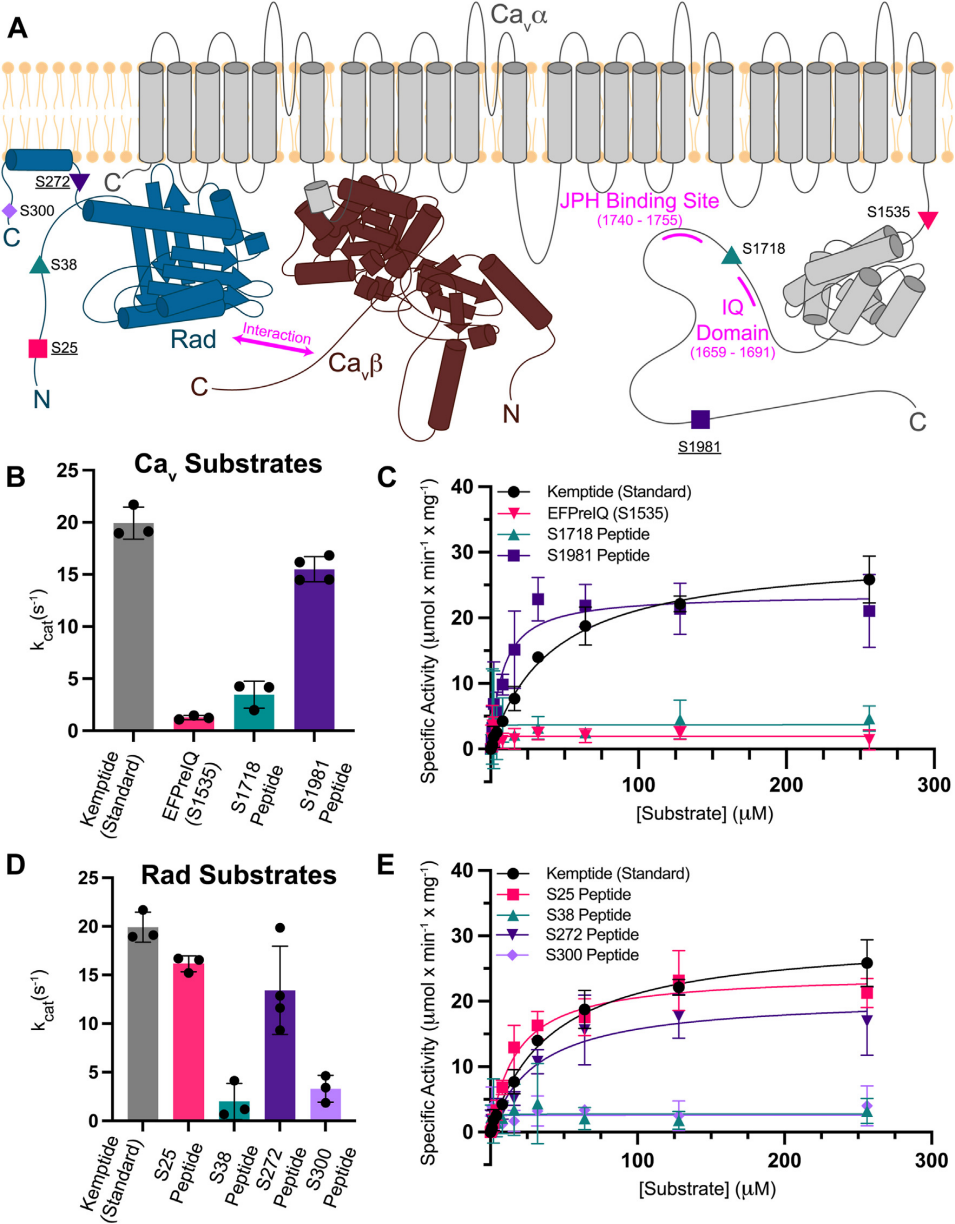
**Kinetics of PKAc phosphorylation of Ca**_**V**_**, Rad, and Kemptide substrates.***A*, a schematic of the membrane topology of the Ca_V_1.2 channel (α_1c_ subunit in *gray* and β subunit in *brown*) and nearby Rad (*dark blue*) with proposed PKA target sites. The binding sites of Junctophilin (JPH) and Calmodulin (IQ domain) are shown in *magenta*. The Rad C-terminal aliphatic α helix is depicted as being anchored parallel to the plane of the membrane. The *underlined target sites* were found to be competent PKAc substrates in this study. *B*, the k_cat_ values determined from the nonlinear regressions of kinase assays utilizing: Kemptide (n = 3, *black*), EFPreIQ (Ser1535) (n = 3, *pink*), Ser1718 peptide (n = 3, *teal*), and Ser1981 peptide (n = 4, *purple*) are shown as a *bar graph*. *C*, the average specific activity values of PKAc-mediated phosphorylation measured at various concentrations of the same substrates as in *panel B*. The *solid curves* depict the nonlinear regression fitted to the data points based on a Michaelis–Menten equation. *D*, the k_cat_ values determined from nonlinear regression fitting of kinase assays utilizing Kemptide (n = 3, *black*) and Rad peptides: Ser25 peptide (n = 3, *pink*), Ser38 peptide (n = 3, *teal*), Ser272 peptide (n = 4, *purple*), and Ser300 peptide (n = 3, *violet*) are shown as a *bar graph*. *E*, the average specific activity values of PKAc-mediated phosphorylation measured at various concentrations of the same substrates as in *panel D*. In panels *B*–*D*, all error bars represent SDs. PKAc, catalytic subunit of PKA.

As Ca^2+^ is a potent intracellular second messenger, fine-tuning the activity of Ca_V_1.2 through phosphorylation plays an important role in various physiological events (10, 11, 12). Initial studies with cAMP-dependent PKA have focused on direct phosphorylation of the pore-forming α_1c_ subunit, with three main proposed sites in the C-terminal cytoplasmic tail: Ser1535, located in a short linker between the last transmembrane helix and the EF-hand domain; Ser1718, in a disordered region between the binding sites for Calmodulin and Junctophilin; and Ser1981, located further downstream, also in a disordered region (Fig. 1*A*). All three sites have been proposed to be functionally relevant. For example, phosphorylation of Ser1535 was shown to result in increased channel openings (13). Similarly, preventing phosphorylation of Ser1718 seemed to reduce channel currents in cardiomyocytes (14). Ser1981 phosphorylation was shown to cause dissociation of the β_2_ adrenergic receptor from the channel (15). Later studies showed that phosphorylation of Ser1981 also augmented Ca^2+^ currents, at least in smooth muscle cells (16) and neurons (17, 18), but not in cardiomyocytes (19).

In contrast with this, another study investigated the augmentation of Ca_V_1.2 currents in cardiac myocytes by mutating all conserved Ser and Thr residues in Ca_V_1.2 (20). The study found that such mutant Ca_V_1.2 channels were still responsive to PKA, indicating that the mechanism for augmentation of currents, at least in cardiac myocytes, is very different. Proximity proteomics demonstrated that Rad, a small GTPase that inhibits Ca_V_s, is enriched in the cardiac Ca_v_1.2 microenvironment but is depleted during β-adrenergic stimulation (21). Together with the finding that Rad is also a direct PKA target, and that its phosphorylation is required for the effect, a model was proposed whereby phosphorylation of Rad leads it to dissociate from Ca_V_1.2, relieving inhibition and thus causing an augmentation of currents (22). In agreement with this, mice lacking four PKA target sites in Rad show a drastic reduction in β-adrenergic–mediated changes in cardiac contractility and in exercise capacity (23).

Although some of the studies seem at odds, it is possible that the precise mechanism of augmentation of Ca_V_1.2 currents is cell type–dependent. Recent studies have also suggested that there are Rad-dependent and Rad-independent effects, both in heterologous expression systems (24) and in cardiac myocytes (25).

Despite the abundance of functional experiments, true characterization of the enzymatic parameters for each target site, as well as any quantitative binding analyses, have been lacking. Knowledge of this would help establish which sites within Ca_V_1.2 and Rad are preferred substrates for PKA and are thus more likely to be phosphorylated initially. Here, we made use of individual peptides that recapitulate the three proposed Ca_V_1.2 and four Rad phosphorylation sites (Table 1). Using isothermal titration calorimetry (ITC) and enzyme kinetics, we show that there are two tiers of substrates, and X-ray crystallographic analysis with the best target site within Ca_V_1.2 shows that PKA has an intrinsic plasticity to engage different target residues.

**Table 1:** Overview of peptides used in this study

Substrate (UniProt ID)	Phosphorylation site	Peptide/substrate sequence^a^	Residue range
Kemptide	N/A	LRRA**S**LG	1–7
Human Ca_V_1.2 (UniProt: Q13936-1)	S1981	RGFLRSASLGRRA**S**FHL	1968–1984
	S1981 (R1979A)	RGFLRSASLGR**A**A**S**FHL	1968–1984
	S1981 (F1982A)	RGFLRSASLGRRA**SA**HL	1968–1984
	S1981 (L1971A)	RGF**A**RSASLGRRA**S**FHL	1968–1984
	S1981 (S1975R)	RGFLRSA**R**LGRRA**S**FHL	1968–1984
Rabbit CaV1.2 (UniProt: P15381-1)	S1928	KGFLRSASLGRRA**S**FHL	1915–1931
Human Ca_V_1.2 (UniProt: Q13936-1)	S1718	RTLHDIGPEIRRAI**S**GDL	1704–1721
Rabbit CaV1.2 (UniProt: P15381-1)	S1700	RTLHDIGPEIRRAI**S**GDL	1686–1703
Human Ca_V_1.2 (UniProt: Q13936-1)	S1535	DYLTRDW**S**ILG	1528–1538
Human Ca_V_1.3 (UniProt: Q01668-1)	S1475 (S1535)^b^	SNADYLTRDW**S**ILG…^a^	1468–1598 (1528–1658)^b^
Rabbit CaV1.2 (UniProt: P15381-1)	S1517	DYLTRDW**S**ILG	1510–1520
Human Rad (UniProt: Q6PGA2-1)	S25	GAGRERDRRRG**S**TPW	14–28
	S38	WGPAPPLHRR**S**MP	28–40
	S272	ARRQAGTRRRE**S**LGK	261–275
	S300	SRKMAFRAKSK**S**CHD	289–303

N/A: not applicable.

In each peptide sequence, the previously proposed phosphorylation site is underlined and bolded.

a Since a short peptide containing the S1475 site could not be obtained, we used a larger construct from Ca_v_1.3 that includes the downstream EF-hand. The sequence around S1475 (S1535 in Ca_V_1.3) is identical in Ca_V_1.2. The first three residues “SNA” are part of a cloning artefact (see Materials and methods). The construct originates from Ca_V_1.3, and the sequence shown in the table is identical in Ca_V_1.2.

b Ser1475 in Ca_V_1.3 corresponds to Ser1535 in Ca_V_1.2. The numbers in brackets correspond to the Ca_V_1.2 sequence.

## Results

We relied on the human Ca_V_1.2 isoform (NCBI NP_955630) for this study. As many previous reports have often made use of rabbit Ca_V_1.2, we provide a sequence alignment of the C-terminal region in Fig. S1, indicating that the proposed PKA target sites and the flanking residues are conserved in these isoforms. For Rad, the mouse sequence (NCBI NP_062636.2) was chosen rather than human, as previous experiments suggesting the importance of Rad were carried out with mice (21).

### Ser1535 and Ser1718 on Ca_V_1.2 are poor PKA substrates compared to Ser1981

We used enzyme kinetics to investigate the activity of catalytic subunit of PKA (PKAc) toward the three target sites within the C terminus of Ca_V_1.2. We performed discontinuous kinase assays using an ADP-GloMax Assay kit. As a positive control, we used Kemptide, a frequently used reference peptide that is readily phosphorylated by PKA (Table 1, Fig. S2). The kinetic properties we determined are within the range of values previously reported for Kemptide, indicating that our assay accurately captures the expected properties of PKAc phosphorylation (Table 2) (Fig. 1) (26, 27, 28, 29).

**Table 2:** Michaelis–menten kinetic parameters of PKAc with Kemptide, Ca_V,_ and Rad substrates

Substrate (sample size)	k_cat_ (s^−1^)	K_m_ (μM)	k_cat_/K_m_ (s^−1^ × μM^−1^)
Kemptide (n = 3)	19.0 ± 1.5	42.6 ± 2.4	0.47 ± 0.05
EFPreIQ (S1535) (n = 3)	1.3 ± 0.2	ND	ND
S1718 peptide (n = 3)	3.4 ± 1.3	ND	ND
S1981 peptide (n = 4)	15.5 ± 1.2	8.1 ± 2.5	1.9 ± 0.6
Rad S25 peptide (n = 3)	16.2 ± 0.8	19.0 ± 6.7	0.9 ± 0.3
Rad S38 peptide (n = 3)	2 ± 1.9	ND	ND
Rad S272 peptide (n = 4)	13.4 ± 4.5	29.9 ± 10.1	0.4 ± 0.2
Rad S300 peptide (n = 3)	3.3 ± 1.4	ND	ND
Ca_V_1.2 S1981 peptide mutants:			
R1979A peptide (n = 3)	1.1 ± 0.1	ND	ND
F1982A peptide (n = 3)	13.1 ± 0.5	9.6 ± 1.7	1.4 ± 0.2
L1971A peptide (n = 3)	19.7 ± 1.3	9.7 ± 2.4	2.0 ± 0.5
S1975R peptide (n = 3)	7.5 ± 0.9	6.2 ± 2.7	1.2 ± 0.5

Errors reported as SD.

ND: Not determined due to inaccurate K_m_ as a result of poor fitting.

PKAc, catalytic subunit of PKA.

For the Ser1981 and Ser1718 sites, we used synthetic peptides (Table 1, Fig. S2). As short peptides containing Ser1535 proved insoluble, we instead opted for a longer construct that also contains the downstream EF-hand domain. The construct corresponding to the Ca_V_1.2 sequence failed to purify, so we instead used the corresponding region in Ca_V_1.3, which could be readily expressed and purified. Importantly, the region around the proposed target site (corresponding to residue Ser1475 in human Ca_V_1.3, Table 1) is identical in both isoforms (Fig. S1).

The Ser1981 peptide is a competent substrate of PKAc, with a k_cat_/K_m_ = 1.9 ± 0.6 s^−1^ × μM^−1^, and is a more robust substrate than the commonly used reference Kemptide (k_cat_/K_m_ = 0.47 ± 0.05 s^−1^ × μM^−1^) (Table 2) (Fig. 1, *B* and *C*). In contrast, the Ser1718 peptide and Ser1535 mimicking construct are poorer substrates, displaying comparatively lower catalytic activities (Table 2) (Fig. 1, *B* and *C*). An accurate K_m_ value cannot be calculated as the low catalytic activity at lower substrate concentrations does not allow an accurate fitting. However, activity is clearly present above background, which was subtracted from all data. As the enzymatic activity leads to a stable Vmax, it becomes saturated, allowing us to obtain a reasonable estimate of the k_cat_ values, corresponding to 3.4 ± 1.3 s^−1^ (Ser1718 peptide) and 1.3 ± 0.2 s^−1^ (Ser1535 construct) (Table 2) (Fig. 1*C*). We note that these values are less reliable than for the Ser1981 peptide but can conclude that Ser1981 is the preferred PKA substrate among the three proposed target sites within Ca_V_1.2.

In a physiological context, it is possible that Ser1718 and Ser1535 are only significantly phosphorylated by PKA during prolonged β-adrenergic signaling, whereas Ser1981 can already become phosphorylated during shorter β-adrenergic stimulation.

### Ser25 and Ser272 on Rad are preferentially targeted for PKA-mediated phosphorylation

Due to the pivotal role of Rad in β-adrenergic signaling of the heart, we conducted kinase assays with four Rad-derived peptides to determine which sites are preferentially phosphorylated. All four putative PKA sites are in predicted intrinsically disordered regions or flanked by secondary structural elements. Both the Ser25 (k_cat_/K_m_ = 0.9 ± 0.3 s^−1^ × μM^−1^) and Ser272 (k_cat_/K_m_ = 0.4 ± 0.2 s^−1^ × μM^−1^) peptides display kinetic properties comparable to Kemptide (k_cat_/K_m_ = 0.47 ± 0.05 s^−1^ × μM^−1^) (Table 2) (Fig. 1, *D* and *E*). In contrast, the Ser38 and Ser300 peptides are poor substrates, with k_cat_ values of 2.0 ± 1.9 s^−1^ (Ser38) and 3.3 ± 1.4 s^−1^ (Ser300), but no reliable K_m_ values could be obtained (Table 2) (Fig. 1, *D* and *E*).

Based on these data, Ser25 and Ser272 seem to form the main targets for PKA in Rad and are likely to become phosphorylated first. Ser38 and Ser300 may only become phosphorylated during longer β-adrenergic stimulation. Comparing the data across both the Ca_V_1.2 and Rad peptides, the Ser1981 site provides the best substrate.

### PKAc binds with micromolar affinity to Ser1981 of Ca_V_1.2 and Ser25 and Ser272 of Rad

**Figure 2:**
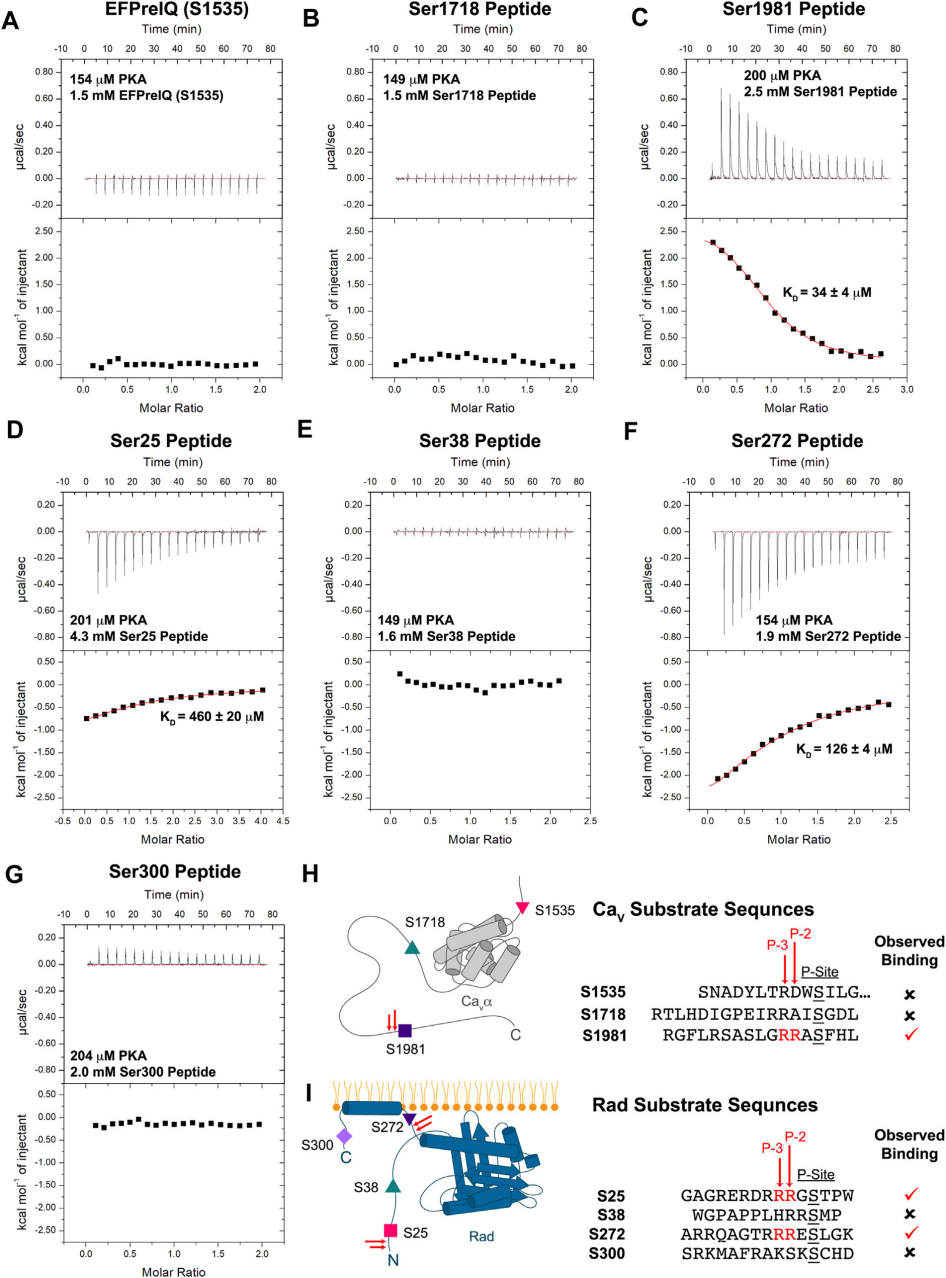
**ITC Experiments of PKAc:AMP-PNP Binding to Ca**_**V**_**and Rad Substrates.** Representative isotherms from ITC experiments titrating (*A*) 1.5 mM EFPreIQ (S1535); (*B*) 1.5 mM S1718 peptide; (*C*) 2.5 mM Ser1981 peptide; (*D*) 4.3 mM Ser25 peptide; (*E*) 1.6 mM Ser38 peptide; (*F*) 1.9 mM S272 peptide; and (*G*) 2 mM Ser300 peptide into 149 to 204 μM of PKAc in the presence of 0.5 mM AMP-PNP and 5 mM MgCl_2_. A dissociation constant (K_d_) was determined based on n = 3 replicates for reactions where binding was detected. Errors correspond to SDs. A schematic illustrating a summary of the results of the isothermal titration calorimetry experiments utilizing (*H*) Ca_V_ substrates or (*I*) Rad substrates. Substrates with arginine residues at the P-3 and P-2 positions are indicated using two *red arrows*. ITC, isothermal titration calorimetry; PKAc, catalytic subunit of PKA.

As an independent means to assess which substrate peptides are preferred by PKAc, we used ITC to determine the inherent affinities for the various peptides. ITC also provides the enthalpy and entropy changes upon binding and can thus help provide a rationale for different affinities. The ITC experiments were performed using excess Mg^2+^ (5 mM) and 0.5 mM adenylyl-imidodiphosphate (AMP)-PNP (a nonhydrolyzable ATP analog) (Fig. 2).

We could not detect any heat signals indicative of binding when using the Ser1535 mimicking construct (Fig. 2*A*) or with the Ca_V_1.2 Ser1718 peptide (Fig. 2*B*). We only detected binding when titrating the Ca_V_1.2 Ser1981 peptide into PKAc (K_d_ = 34 ± 4 μM) (Fig. 2*C*) (Table 3). This confirms the enzyme kinetic findings indicating that the Ser1981 peptide is the preferred PKA substrate among the three. Of note, the binding to this peptide is endothermic, and is thus entirely driven by positive changes in entropy (ΔH = 13 ± 1 kJ × mole^−1^, -TΔS = −36 ± 2 kJ × mole^−1^).

**Table 3:** Thermodynamic binding properties of PKAc:AMP-PNP to substrates

Substrate (replicates)	$\bar{K_{D}\;}$ (μM)	$\bar{N\;}$	$\bar{\Delta G\;}$ (kJ × mole^−1^)	$\bar{\Delta H\;}$ (kJ × mole^−1^)	$-\bar{T\Delta S\;}$ (kJ × mole^−1^)
Ca_V_1.2 S1981 peptide (n = 3)	34 ± 4	0.98 ± 0.05	−23.7 ± 0.2	13 ± 1	−36 ± 2
Ca_V_1.2 S1718 peptide (n = 1)	ND	ND	ND	ND	ND
Ca_V_1.3 EFPreIQ (S1535) (n = 1)	ND	ND	ND	ND	ND
Rad S25 peptide (n = 3)	460 ± 20	0.83 ± 0.06	−17.7 ± 0.1	−10.9 ± 0.3	−6.8 ± 0.2
Rad S38 peptide (n = 2)	ND	ND	ND	ND	ND
Rad S272 peptide (n = 3)	126 ± 4	0.98 ± 0.05	−20.70 ± 0.08	−17 ± 1	−4 ± 1
Rad S300 peptide (n = 1)	ND	ND	ND	ND	ND
Ca_V_1.2 S1981 peptide mutants:					
R1979A peptide (n = 3)	ND	ND	ND	ND	ND
F1982A peptide (n = 3)	147 ± 26	0.99 ± 0.4	−20.4 ± 2	10.3 ± 1.2	−30 ± 1.7
L1971A peptide (n = 3)	18 ± 0.4	1.06 ± 0.025	−25.2 ± 0.7	11 ± 0.4	−36 ± 0.5
S1975R peptide (n = 3)	532 ± 880	1.08 ± 0.07	−21.4 ± 9	14 ± 1.7	−36 ± 9

Peptides were titrated into the cell containing PKAc. Measurements were performed at 4 °C in 50 mM Hepes (pH 7.4), 150 mM KCl, 5 mM MgCl_2_, and 2 mM TCEP. Errors reported as SDs. ND: Not determined due to unobservable binding or poor fit.

AMP-PNP, adenylyl-imidodiphosphate; PKAc, catalytic subunit of PKA; TCEP, tris(2-carboxyethyl)phosphine hydrochloride.

For the two Rad peptides within the N-terminal tail of the protein, we detected binding for the Ser25 peptide (K_d_ = 460 ± 20 μM) (Fig. 2*D*) (Table 3), but not for the Ser38 peptide (Fig. 2*E*) (Table 3). For the C-terminal peptides, we detected binding when using the Ser272 peptide (K_d_ = 126 ± 4 μM) (Fig. 2*F*) (Table 3), but not when using the Ser300 peptide (Fig. 2*G*). Thus, this confirms the enzymatic experiments suggesting the Ser25 and Ser272 peptides are the preferred PKA substrates (Fig. 2*H*). In contrast to the Ser1981 peptide, binding of both peptides to PKA is exothermic. The binding appears driven by both favorable enthalpy and entropy changes for both the Ser25 peptide (ΔH = −10.9 ± 0.3 kJ × mole^−1^, -TΔS = −6.8 ± 0.2 kJ × mole^−1^) and for the Ser272 peptide (ΔH = −17 ± 1 kJ × mole^−1^, -TΔS = −4 ± 1 kJ × mole^−1^) peptides (Table 3).

Overall, PKA thus has the highest affinity for the Ca_V_1.2 Ser1981 peptide, followed by Rad Ser272 and Rad Ser25. However, it is important to note that, given the weak affinities, there are substantial errors in the precise K_d_ measurements for the Rad peptides. Technically there is even more error in the stiochiometry as the C values are quite low (C=nx[cell]/KD). Although ITC failed to detect interactions with the other peptides, this does not imply that there is no binding at all, but that it is simply too weak to be detectable *via* this method.

### Structures of ApoPKAc, AMP-PNP PKAc, and PKAc-Ser1981 ternary complexes

Given our findings that the Ca_V_1.2 Ser1981 peptide, Rad Ser25 peptide, and Rad Ser272 peptides are all competent PKA substrates, we attempted to capture crystal structures of PKAc ternary complexes with each of these substrates to help better understand PKA substrate specificity. Upon purifying PKAc, we obtained two major peaks (peak 1 and peak 2) on the ion change column that correspond to different degrees of phosphorylation (Fig. S3). These gave rise to two different structures of ternary complexes of PKAc bound to both AMP-PNP and the Ca_V_1.2 Ser1981 peptide at 2.85Å and 2.99Å resolutions, respectively (Table S1). Complex 1 (C1), derived from peak 1, is likely phosphorylated at three sites on PKAc, whereas complex 2 (C2), derived from peak 2, contains two phosphorylated sites. Thus far, complexes with the Rad peptides escaped crystallization, likely a result of their weaker affinity for PKAc. We also collected a dataset for PKAc in the presence of the Ser1718 peptide. This 2.75 Å dataset contains four PKAc molecules in the asymmetric unit referred to here as Apo/AMP-PNP PKAc, as it lacks density for the Ser1718 peptide. Three out of the four molecules in the asymmetric unit are in the apo state (chain C: ApoPKAc1, chain F: ApoPKAc2, and chain H: ApoPKAc3) and one is bound to AMP-PNP (chain D). Altogether, our structures depict PKAc in different catalytic stages and conformations.

### Dynamic nature of the PKAc catalytic cycle

**Figure 3:**
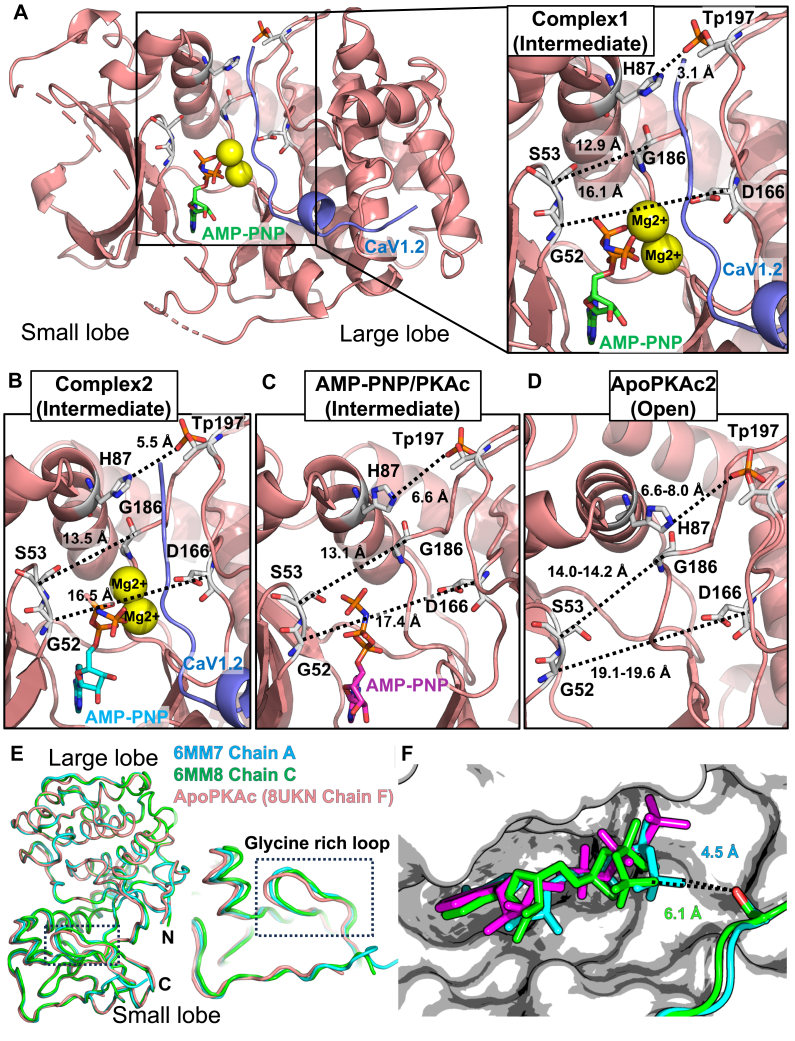
**Conformational differences of the PKAc structures.***A*, overview of PKAc + Ser1981 peptide complex 1 (PDB: 8UKP), highlighting AMP-PNP in *green*, Ca_V_1.2 peptide in *blue*, and Mg^2+^ in *yellow*. Inset shows residues/distances used to classify the open/intermediate/closed state of PKAc molecules. *B*, similar view for PKAc + Ser1981 peptide complex 2 (PDB: 8UKO), (*C*) AMP-PNP:PKAc (PDB: 8UKN, chain *D*), and (*D*) the ApoPKAc2 structure (PDB: 8UKN, chain *F*). The distances listed are for ApoPKAc listed as the range of distances observed in all three ApoPKAc1-3 molecules (chains *C*, *F*, and *H*). PKAc molecules are depicted as *cartoons* and *sticks*. The *sticks* are colored according to the atom type: carbon: *white*; oxygen, *red*; nitrogen, *blue*; phosphorus, *orange*. The Ser1981 peptide is colored *blue* as a *cartoon representation*. Mg^2+^ ions are depicted as *yellow spheres*. The AMP-PNP molecules are displayed as *sticks* with carbon atoms colored *A*, *green*, *B*, *cyan*, and *C*, *magenta*. *E*, two PKAc structures from RyR2 ternary structures with the lowest RMSDs and highest similarity score (Z-score) calculated *via* the DALI server 2 are depicted as *cartoons* and are overlaid the ApoPKAc2 structure (PDB: 8UKN, chain *F*). The glycine-rich loop is highlighted using a dashed black box. *F*, differences in the substrates of AMP-PNP:PKAc, complex 1, and complex 2 molecules are shown. The PKAc molecule is shown as *ribbons* and colored according to the previous scheme. The substrates (AMP-PNP and Ser1981 peptide) are colored *magenta* (AMP-PNP:PKAc), *green* (complex 1, PDB: 8UKP), and *cyan* (complex 2, PDB: 8UKO). The AMP-PNP molecules are shown as *sticks*. The Ser1981 side chain is shown as *sticks* with its hydroxyl group colored *red*. AMP, adenylyl-imidodiphosphate; PKAc, catalytic subunit of PKA.

The PKAc conformations can be classified as being in the open, intermediate, or closed states. This classification is based on the distances of the C_α_ atoms of Gly52 and Asp166, the C_α_ atoms of Ser53 and Gly186, the Nε2 atom of His87 and closest oxygen in pThr197 side chain, and the backbone oxygen of Glu170 and the hydroxyl group of Tyr330 (26). Based on these criteria, our Apo structures (apoPKAc) depict an open state of the kinase, whereas the AMP-PNP and ternary complexes correspond to different intermediate states (Fig. 3, *A*–*D*). The distances between these residue pairs decrease in length in the order: ApoPKAc3 > ApoPKAc1 > ApoPKAc2 > AMP-PNP PKAc > C2 > C1 (Table S2). Thus, these structures represent various states of the kinase, starting from the open/unbound state to a fully bound, intermediate state prior to subsequent substrate turnover. C1 and C2 correspond to different degrees of phosphorylation of PKAc. Both are phosphorylated at Thr197 and Ser338, but C1 is also phosphorylated at Ser139. None of these three sites are in direct contact with the substrate peptide, are solvent exposed, and Ser139 in particular is > 20 Å away from the nearest Ca_V_1.2 residue. Thus, it is unlikely that the phosphorylation of PKAc Ser139 directly underlies any differences observed in C1 and C2.

Most notably the ApoPKAc2 molecule adopts a conformation distinct from the other two ApoPKAc molecules in the asymmetric unit. A DALI search (30) of ApoPKAc2 yields top hits of some of our previously published PKAc:RyR2 ternary structures with RMSD values of 0.6 to 0.9 Å (Fig. S4). The main difference observed between these structures and ApoPKAc2 is the glycine-rich loop (residues 47–56) (Fig. 3*E*). This is expected, as the stable positioning of the loop is largely dependent on the presence of a bound nucleotide and can vary greatly in ApoPKAc structures (31). Nevertheless, the ApoPKAc2 molecule adopts a conformation that most closely resembles a substrate-bound state. This lends support to the conformational selection model as this conformation of PKAc appears to be thermodynamically stable and can be sampled both in the presence and absence of substrate (32, 33, 34). The dynamic nature of these structures extends to the active site, as the γ-phosphate of the AMP-PNP molecule adopts various positions in our structures (Fig. 3*F*). Interestingly, despite C1 representing a more closed state than C2, in the latter the γ-phosphate is more readily primed for phosphoryl transfer, indicated by its shorter distance from the Ser1981 hydroxyl group (Figs. 3*F*, S5). Altogether these observations highlight the dynamic nature of the enzyme both in the presence and absence of substrate.

### PKAc specificity to Ser1981

**Figure 4:**
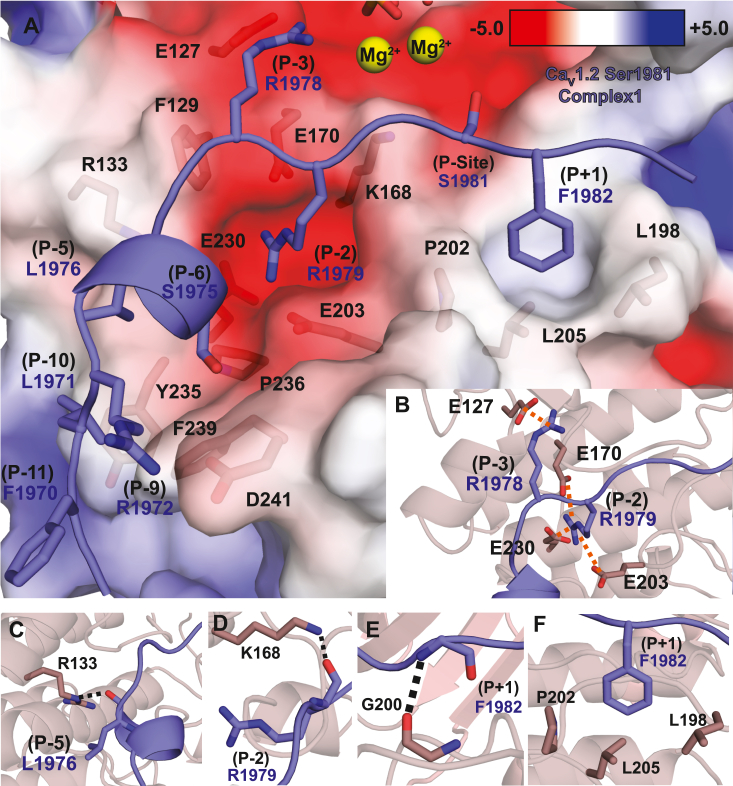
**Critical binding determinants for PKAc specificity to the Ca**_**V**_**1.2 Ser1981 peptide.** Residues involved in enzyme-peptide recognition are shown as *sticks*. The Ca_V_1.2 peptide is shown in *purple* and PKAc in *salmon/dark salmon* (small lobe/large lobe). Nitrogen atoms are colored in *red* and oxygen in *blue*. *A*, a surface representation of the active site of PKAc is colored according to electrostatic potential. Residues involved in vdW and electrostatic interactions are shown as *sticks*. Mg^2+^ ions are depicted as *yellow spheres*. *B*, an electronegative pocket is formed by E170, E203, and E230 in which the R1979 side chain at the P-2 position of the S1981 peptide slots into forming salt bridging interactions with E230, E170, and E203. The R1978 in the P-3 position forms a salt bridge with E127. The salt bridges are depicted as *orange dotted lines* and are drawn from charge center of one side chain to the other. *C*, the P-5 leucine of the S1981 peptide forms a hydrogen bond with R133 through its backbone oxygen atom (*dashed line*). *D*, the main chain oxygen of R1979 forms a hydrogen bond with the K168 side chain (*dashed line*). *E*, the oxygen backbone atom of G200 forms a hydrogen bond with the backbone nitrogen atom of F1982 (*dashed line*). *F*, a hydrophobic pocket formed by L198, P202, and L205 is occupied by the F1982 side chain of the peptide forming extensive hydrophobic contacts. PKAc, catalytic subunit of PKA.

Our PKAc:substrate ternary complexes contain strong density for residues Gly1969–Phe1982 of the Ser1981 peptide (Figs. S5 and 4A). As evident from our kinase assays and ITC experiments, the two Arginine residues at the P-2 and P-3 positions are critical for PKA substrate specificity, as only the peptides containing these showed detectable binding. These residues have previously been found to be important for interactions with both substrates and inhibitory peptides (26, 32, 35, 36). The Arginines mediate multiple interactions with PKAc (Fig. 4*B*). The P-3 Arg side chain participates in a salt bridge interaction with PKAc Glu127. The P-2 Arg side chain fits into an electronegative pocket of the active site formed by the side chains of Glu170, Glu203, and Glu230, forming salt bridges with all three. Outside of these Arginine residues, the Ser1981 peptide engages in multiple hydrogen bonding interactions. The Arg133 side chain of PKAc hydrogen bonds with the backbone oxygen of the P-5 Leu1974 (Fig. 4*C*). In addition to its side chain, the backbone oxygen of the P-2 Arg1979 also forms a hydrogen bond with the side chain of Lys168 of PKAc (Fig. 4*D*). The P + 1 Phe1982 nitrogen backbone atom forms a hydrogen bond with the Gly200 oxygen backbone atom (Fig. 4*E*). In addition to these interactions, both PKAc:AMP-PNP:Ca_V_1.2 Ser1981 peptide complexes present an interaction mode not yet seen in PKAc:substrate complexes. The P + 1 phenylalanine side chain fits into a hydrophobic pocket of the enzyme, formed by the side chains of PKAc Leu198, Pro202, and Leu205 (Fig. 4*F*). Finally, extensive van der Waals interactions are observed through the N-terminal portion of the peptide (Fig. 4*A*) involving Leu1971 (P-10), Arg1972 (P-9), Ser1975 (P-6), Leu1974 (P-5), and Gly1977 (P-4) with various PKAc residues (Table S3 and Figs. S6, S7) with a total buried surface area of 257.2 Å^2^ in C1 and 210.21 Å^2^ in C2.

**Figure 5:**
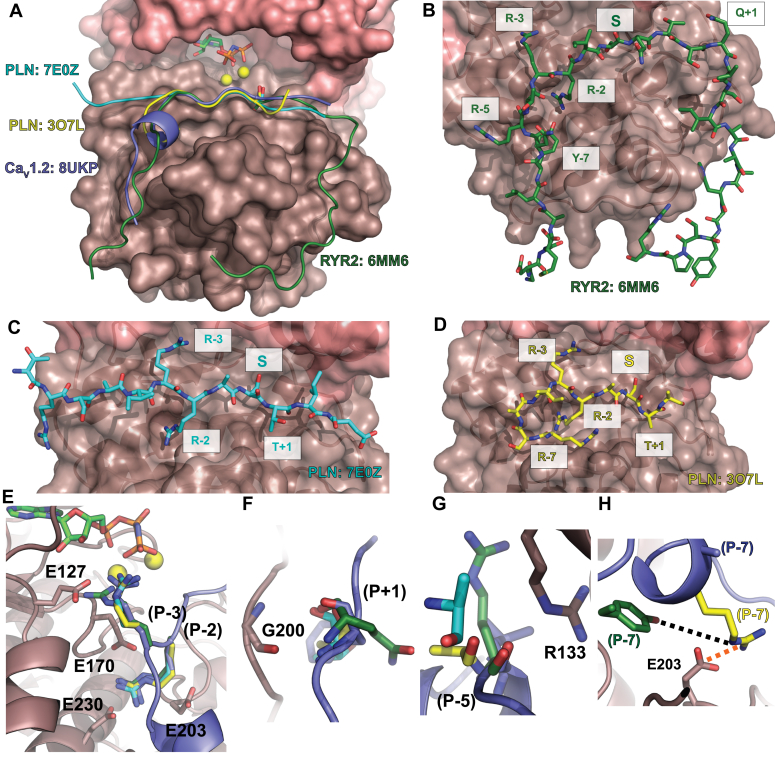
**Comparison of PKAc substrate interactions to Ca**_**V**_**1.2.***A*, surface representation of PKAc of complex 1 (PDB: 8UKP, this study) with the small lobe colored in *salmon* and large lobe colored in *dark salmon*. AMP-PNP are represented in *sticks* with the Ca_V_1.2 Ser1981 peptide shown in *blue cartoon*. Substrates from other PKAc ternary structures are also shown in *cartoon representation*: RyR2 phosphorylation domain (*green*) (PDB: 6MM6), and two phospholamban structures (*cyan* and *yellow*) (PDB: 7E0Z and 3O7L, respectively). *B*–*D*, residues important for substrate recognition and specificity for each respective peptide are labeled. For each peptide, the carbons are colored as the *cartoons* in *panel A*, and with oxygen atoms in *red* and nitrogen atoms in *blue*. *E*, P-2 and P-3 arginine residues are present in all structures and positioned similarly in all substrates and interacts through various salt bridges and hydrogen bonds. *F*, the backbone atoms of G200 of PKAc interacts with the P + 1 backbone in all PKAc ternary structures. *G*, RyR2 and Ca_V_1.2 interact with R133 through the backbone oxygen of the P-5 residue. The equivalent residue in PLN does not. *H*, RyR2 and PLN (PDB: 3O7L but not 7E0Z) P-7 residue side chain interacts through a hydrogen bond (*black dotted line*) and salt bridge (*orange dotted line*), respectively, to E203. Ca_V_1.2 harbors an alanine at this position and thus lacks a hydrogen bond donor/acceptor to engage in similar interactions. PKAc, catalytic subunit of PKA; PNL, phospolamban.

Although many structures of PKAc have been reported, only two other ternary PKAc:substrate complexes are available. Figure 5 compares the binding of Ca_V_1.2 (Ser1981 peptide) with RyR2 and phospolamban (PLN) (Fig. 5, *A*–*D*), highlighting the divergence in the interactions. In all structures, the P-2 and P-3 Arginine side chains are at near identical positions (Fig. 5*E*). The backbone nitrogen atom of the P + 1 atom of each substrate engages in a hydrogen bond with Gly200 backbone oxygen atom of PKAc (Fig. 5*F*). Although all substrates engage in extensive van der Waals interactions at the N-terminal portion of the substrate, they utilize different residues. The P-5 backbone hydrogen bonding interaction with Arg133 observed in Ca_V_1.2 is also present in RyR2 but not in PLN (Fig. 5*G*). The Ca_V_1.2 Ser1981 peptide differs from both PLN and RyR2 in the usage of a P-7 residue (Fig. 5*H*). In Ca_V_1.2, this residue is an alanine that does not form specific interactions, in contrast with RyR2 where a Tyr residue packs into a shallow hydrophobic groove of PKAc and forms a hydrogen bond with Glu203. In PLN (PDB: 3O7L), an Arg at this position either forms a salt bridge with Glu203 (PDB: 3O7L) or forms a hydrogen bond with the backbone oxygen atom of Arg134 (PDB: 7E0Z) (Fig. S7). Thus, the precise interactions differ in both the N-terminal and C-terminal regions of the substrate peptide. A summary of all interactions is also shown in Fig. S7 as a LIGPLOT 2D diagram (37).

### Assessing the contribution of key residues in the Ser1981

**Figure 6:**
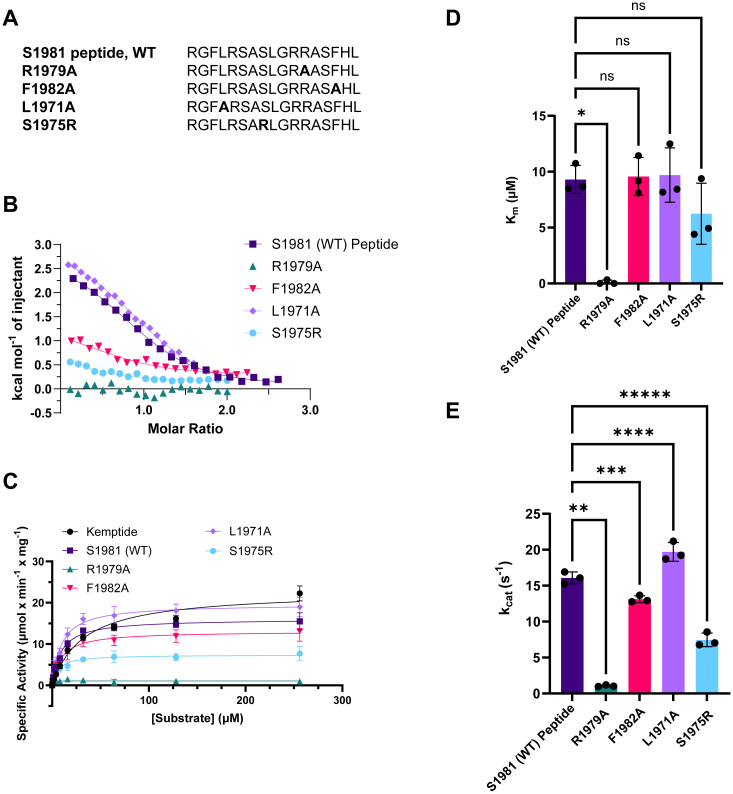
**Effect of mutations in the Ca**_**V**_**1.2 Ser1981 peptide.***A*, sequences of the various mutant peptides used. *B*, superposition of representative integrated ITC data, along with their fits, for the various peptides. *C*, the average specific activity values of PKAc-mediated phosphorylation measured at various concentrations of Kemptide (*black*) and the WT and mutant Ser1981 peptides. n = 3 for each experiment. *D*, average K_m_ values for the WT and mutant peptides. ∗*p* = 0.0005 (one-way ANOVA; F = 14.11). *E*, average k_cat_ values for the same peptides. ∗∗*p* < 0.0001; ∗∗∗*p* = 0.0051; ∗∗∗∗*p* = 0.0012; and ∗∗∗∗∗*p* < 0.0001 (one-way ANOVA; F = 229.9). In panels C- E, all error bars represent SDs. PKAc, catalytic subunit of PKA; ITC, isothermal titration calorimetry.

To further explore the importance of distinct residues in the Ser1981 peptide, we used mutant versions to test their contribution to binding and enzymatic activity (Fig. 6*A*).

We first tested the importance of Phe1982 at the P + 1 site, which forms extensive hydrophobic interactions with PKA. Upon truncating the side chain to Ala, there is a >3-fold reduction in affinity (Table 3, Fig. 6*B*), indicating that this residue makes significant contributions to the binding. However, the ability of the mutant peptide to be phosphorylated by PKAc is only mildly affected, with a ∼26% reduction in k_cat_/K_m_ (Table 2, Fig. 6, *C*–*E*).

Next, to probe the importance of the Arg at the P-2 site, we introduced the R1979 A mutation. We could no longer detect any interaction using ITC, and the enzymatic activity was very low, so that a reliable K_m_ value could not be obtained. This highlights the importance of the P-2 residue, which is absent in the Ca_V_1.2 Ser1535 and Ser1718 and Rad Ser300 peptides.

We next investigated the role of Leu1971 and its contribution to the P-10 site. Surprisingly, the L1971A peptide has a ∼2-fold increased affinity for PKAc (Table 3). Although there is also an increased k_cat_/K_m_, this effect is not statistically significant. Leu1971 clearly makes interactions with PKA in the crystal structures, which include van der Waals interactions with the charge-carrying guanidinium moiety of Arg133. Possibly, the interaction between a hydrophobic and a charged side chain may prevent otherwise favorable interactions between Arg133 and solvent, leading to a negative contribution to binding affinity that is absent in the L1791A mutant.

So far, all substrates tested have a low affinity for PKAc. Whereas this is expected for an enzyme–substrate interaction, it is in stark contrast with PKI, which is known to bind PKAc with a nanomolar affinity (36). Although there are many differences, PKI utilizes an Arg at the P-6 position, which makes ionic interactions with Glu204 in PKAc. In the Ser1981 peptide, there is instead Ser1975 at this position, whose side chain makes no interaction with PKAc. We therefore introduced the S1975R position, to see whether this would improve the catalytic activity. Interestingly, however, the mutation decreased the affinity for PKAc more than 15-fold (Table 3, Fig. 6), and also decreased k_cat_/K_m_ by 37% (Table 2, Fig. 6*E*). In our crystal structure, Ser1975 is pointing directly to PKAc, and there is no space for a larger Arg residue, which would readily explain the drastically reduced affinity. This suggests that the ability of an Arg at the P-6 position to improve binding to PKAc likely depends on upstream residues that impinge on the main chain conformation and allow alternative interactions. Of note, PLN also contains a Ser at the P-6 position, and in one crystal structure an Arg at P-7 was shown to interact with Glu204 instead (Fig. S8). This further supports the idea that sequences surrounding a particular residue can impact where it can bind within PKAc.

## Discussion

We set out to provide an unbiased analysis of how PKA recognizes various proposed target sites in Ca_V_1.2 and Rad, directly determining binding energies and enzyme kinetic parameters. Although phospho-specific antibodies can be used to detect the degree of phosphorylation in full-length proteins in a physiological context, these do not allow for a direct comparison of different sites, as the signals are dependent on the affinity of the antibodies for their respective binding sites. In this study, we used short peptides that mimic the target sites, since similar experiments with full-length proteins would not allow us to isolate the individual sites.

The results of our kinase and ITC experiments indicate that Ser1981 of Ca_V_1.2 and Ser25 and Ser272 of Rad are most readily phosphorylated by the PKAc α isoform (Fig. 1). Despite numerous reports describing the importance of Arginines at the P-2 and P-3 positions (38), several sites lacking these pairs have been proposed as robust PKA targets in Ca_V_1.2 and Rad. Our data underscore the importance of these Arg residues for substrate recognition, as only the peptides containing these showed significant binding and kinase activity (Figs. 1 and 2) (Tables 2 and 3). Indeed, mutating the Arg at the P-2 site in the Ser1981 peptide almost completely obliterates catalytic activity of PKAc towards this substrate, an effect in stark contrast with other mutations we tested, whose effects were more subtle (Fig. 6*E*). Of note, two additional peptides in this study contain double Arginine motifs upstream of the target Ser: The Ca_V_1.2 Ser1718 peptide at the P-3 and P-4 positions, and the Rad Ser38 peptide at the P-1 and P-2 positions. However, not having these Arginines at the P-2 and P-3 would put the target Ser away from the γ-phosphate of ATP, leading to poor activity. Also, allowing anchoring *via* these two Arginines would put alternative residues in that position: Ile1717 (Ser1718 peptide) and Met39 (Ser38 peptide), which would likely also clash with PKAc in those positions. This explains our inability to detect binding of these peptides to PKAc using ITC.

Even though the other peptides lack the double Arg pair at the P-2 and P-3 sites, our kinase assays still indicate some enzymatic activity, albeit very low. Other factors may also contribute to significant phosphorylation in physiological settings, such as A-kinase anchoring proteins that have been proposed to associate with Ca_V_1.2, thus enhancing the local concentration of substrate sites next to PKA (39, 40, 41, 42, 43). Any membrane localization of PKA would also affect activity and may affect different phosphorylation sites of Rad depending on their relative accessibility levels when Rad is bound to the plasma membrane. Thus, even poor substrates can become significantly phosphorylated *in vivo.* However, this local concentration would also apply to the robust target sites, suggesting the presence of two tiers of substrates. This opens the door for a graded response, whereby short-term β-adrenergic stimulation results in phosphorylation of primarily Ser1981 on Ca_V_1.2 and Ser25 + Ser272 on Rad. Longer, more chronic stimulation, perhaps together with reduced activity of phosphatases, may lead to significant phosphorylation of the additional sites. Such a graded response may also have functional consequences not captured with short-term β-adrenergic stimulation.

The C-terminal tail of Rad contains a polybasic α-helix that is thought to mediate interactions with the plasma membrane, enabling the ability of Rad to inhibit Ca_V_1.2. Ser272 resides in this helix, and previous assays using ^32^P-ATP had suggested it to be the only PKA target in Rad (44). Its phosphorylation thus adds a negative charge that may reduce the affinity for the plasma membrane, resulting in a disinhibition of the channel (22, 45). However, the involvement of the residues in fully disordered regions should not be ignored. It has been previously observed that phosphorylation of intrinsically disordered regions can affect neighboring structured domains (46, 47, 48). In the case of RyR2, for example, a phosphomimetic at site Ser2814 was shown to induce α-helicity (26). Thus, the possibility still exists that phosphorylation of other residues in Rad imparts structural changes.

Recently, it was shown that the N-terminal PKAc sites in Rad (Ser25 and Ser38) are not relevant for augmentation of Ca_V_1.2 currents in cardiac myocytes, but both Ser272 and Ser300 are deemed important (22). Ser272 is the better substrate and would get phosphorylated first, but the question arises whether this may affect the ability of PKAc to phosphorylate Ser300. Longer Rad constructs proved to have reduced solubility and difficulties with purification, and we were thus unable to test this hypothesis. Of note, we previously observed such an effect for a folded domain of RyR2, where a phosphomimetic at Ser2814 increased the catalytic activity of PKAc toward Ser2808, by virtue of changes in the local structure of RyR2. In Rad, however, the Ser272 and Ser300 residues are much further apart, and whether similar crosstalk is possible remains to be tested.

Our study also provides additional insights into substrate recognition by PKAc (Fig. 5). Although the binding of PKA to inhibitory peptides is well documented, structures with physiological substrates have been scarce, likely due to their intrinsically weak affinity for PKA. With the current study included, there are now three enzyme:substrate complexes available for PKAc: 1) the cardiac RyR2 phosphorylation hot spot domain containing Ser2808 (26), 2) PLN (32, 35), and 3) the Ca_V_1.2 C-terminal tail containing Ser1981 (this study). Although some interactions appear conserved among the substrates, there are several distinct differences. For example, an aromatic residue at P-7 in RyR2 is buried into a shallow hydrophobic pocket in PKAc. In contrast, PLN utilizes a salt bridge at this position. In Ca_V_1.2 (Ser1981 site), the P-7 position does not contribute, but instead an aromatic anchor at the P + 1 position provides additional stabilization. Of note, kinase:substrate interactions are typically weak, but the PKA inhibitor PKI binds to PKAc with high affinity (36). Interestingly, PKI makes use of both the P-6 and P + 1 positions, which contributes to its much higher affinity (Fig. S9).

To our knowledge, no physiological PKAc substrate displays higher than mid-micromolar affinity. This relatively poor affinity of PKAc for its substrates is likely important for the catalytic turnover. It has been proposed that tight binding of a substrate could limit turnover rates by prolonging product release (32). We tested several mutants in the Ca_V_1.2 Ser1981 peptide, and found that the L1971A mutation increased the affinity ∼2-fold. However, there was no concurrent increase in k_cat/_K_m_, indicating that improved binding does not imply increased enzymatic activity. This notion was also observed in kinetic assays utilizing a phosphorylation-competent PKI peptide, which binds at nM affinity, yielding a relatively poor substrate when compared head-to-head to Kemptide (36). Additionally, despite being phosphorylation-competent, the PKA regulatory RII subunit binds to PKAc with nM affinity (49, 50) and exhibits “single turnover autophosphorylation” suggested by the nondetectable kinetic activity of the kinase (51, 52). Altogether, these data support the hypothesis that PKAc substrates must balance specificity and affinity to mediate adequate levels of turnover.

Mutations in Ca_V_1.2 have been linked to several disorders, including Brugada, long QT, and Timothy syndromes (53, 54, 55). Mining of the clinvar database (https://www.ncbi.nlm.nih.gov/clinvar/) shows that several sequence variants, found in patients with long-QT, affect residues in the Ser1981 peptide (Table S4). This includes variants in the target serine itself (S1981P, S1981F), as well as the two critical P-2 and P-3 Arg residues (R1978Q, R1979K). Such variants undoubtedly strongly diminish phosphorylation by PKA, through removal of critical contacts. Other variants likely introduce steric hindrance (G1969A, G1977S, G1977D) or restrict the backbone flexibility (S1973P, A1974P). It is currently unknown whether these variants truly cause long-QT and their presence in patients further argues against a role of Ser1981 in augmenting cardiac Ca_V_1.2 currents: if Ser1981 phosphorylation increased currents, then mutations that abolish this would cause a net reduction in depolarizing currents, at least under conditions of β-adrenergic signaling, which would reduce, rather than prolong, the cardiac action potential.

Our work provides insight into the PKA-mediated phosphorylation of Rad elucidating that both Ser25 and Ser272 are phosphorylated by PKA more readily than Ser38 and Ser300. We conclude that the presence of P-2 and P-3 Arginines are critical for PKA specificity whereas other aspects such as backbone atom hydrogen bonding interactions *via* the P-2 and P + 1 with Lys168 and Gly200, respectively, are important as they are observed in all PKAc:substrate structures published to date. Finally, different substrates utilize interactions that are not observed in other substrates exemplifying the plasticity in which PKAc can recognize its substrate. Although we were unsuccessful in cocrystallizing the Rad peptides with PKA, further insights may be obtained from such structures, and it will be of interest to test whether the phosphorylation of these peptides results in changes in secondary structure.

## Experimental procedures

### DNA constructs

A mouse PKAcα (cAMP-dependent kinase catalytic subunit, isoform alpha) (UniProtKB/Swiss-Prot: P05132-1) DNA sequence and a human Ca_V_1.3 DNA sequence were cloned into a modified pET28 vector containing an N-terminal 6xHis tag, maltose-binding protein tag, and tobacco etch virus (TEV) protease cleavage site. The cloned PKAcα sequence consisted of residues 16 to 351 (pET28HMT-PKAcα_16-351_). The Ca_V_1.3 construct consisted of residues 1468 to 1598 (pET28HMT-EFPreIQ) (UniProtKB/Swiss-Prot: Q01668-1) which make up the EF-Hand and PreIQ domain of Ca_V_1.3.

### Recombinant PKAc protein expression and purification

The pET28HMT-PKAcα_16-351_ plasmid was transformed into *Escherichia coli* Rosetta (DE3)pLysS competent cells and plated against chloramphenicol (34 μg/ml) and kanamycin (50 μg/ml) selection for 16 h at 37 °C. Flasks containing autoinduction media (56) were inoculated and grown at 37 °C for 6 h at 180 rotations per minute (RPM) before changing the temperature to 18 °C. The cells were then grown for an additional 64 h before harvesting, in which cell pellets were stored at −20 °C.

All steps in lysis and purification were performed at 4 °C or on ice. Upon thawing, cell pellets were lysed *via* sonication in a lysis buffer containing 20 mM 4-(2-hydroxyethyl)-1-piperazineethanesulfonic acid (Hepes) (pH 7.4), 250 mM KCl, 10 mM imidazole, 10% glycerol, 10 mM MgCl_2_, 1 mM PMSF, 1 mM tris(2-carboxyethyl)phosphine hydrochloride (TCEP), 25 μg/ml DNaseI, and 25 μg/ml lysozyme. The lysed cell contents were pelleted *via* centrifugation (Beckman Coulter, Avanti J-E) at 40,000*g* for 35 min. The soluble lysate was filtered with a 0.45 μm nylon syringe filter and then applied to a HisTrap Fast Flow immobilized metal affinity chromatography (IMAC) column (GE Healthcare Lifesciences) pre-equilibrated with buffer A (20 mM Hepes [pH 7.4], 250 mM KCl). The column was washed with 15 column volumes (CV) of buffer A before eluting using buffer A + 400 mM imidazole. The elution was pooled, spiked with 2 mM EDTA, 2 mg of TEV and dialyzed in dialysis buffer (20 mM Hepes [pH 7.4], 250 mM KCl, and 10 mM β-mercaptoethanol [β-ME]) overnight. The dialyzed sample was then applied to an Amylose Column (New England Biolabs) pre-equilibrated with buffer A. The flowthrough was then collected and applied to a PorosMC column (Thermo Fisher Scientific) pre-equilibrated with buffer A. The flowthrough was collected and dialyzed against 10 mM KH_2_PO_4_ (pH 6.6), 10 mM KCl, and 10 mM β-ME for 3 h. The dialyzed sample was applied to a ResourceS (GE Healthcare) column pre-equilibrated with 10 mM MES (pH 6.6), 10 mM KCl, and 15 mM β-ME and eluted over a 0% to 23% gradient of 10 mM MES (pH 6.6), 1 M KCl, and 15 mM β-ME over 25 CV. Fractions containing recombinant PKAc verified *via* SDS-PAGE were pooled and concentrated to 250 μl using an Amicon concentrator (10 K molecular weight cut-off (MWCO); Millipore). The concentrated sample was then applied to a preparative-grade Superdex200 column (GE Healthcare) pre-equilibrated with ITC/assay buffer (50 mM Hepes [pH 7.4], 150 mM KCl, 5 mM MgCl_2_, and 2 mM TCEP). Fractions containing soluble and monomeric PKAc were then pooled and concentrated to 125 to 300 μM for use in ITC experiments or concentrated to 500 μM, spiked with 30% glycerol, flash-frozen, and stored at −80 °C for later use in kinase assays.

PKAc intended for crystallization was purified using a protocol to separate out the different auto-phosphorylated forms as previously described (33, 57). Briefly, PKAc was dialyzed against 10 mM potassium-phosphate buffer pH 6.3–6.4 plus 20 mM KCl and 10 mM β-ME before cation exchange. PKAc sample was applied to the SP column equilibrated with 15 mM potassium-phosphate buffer pH 6.3, 20 mM KCl plus 10 mM β-ME, and subsequently eluted with a gradient of 2% to 28% of buffer containing an additional 1 M KCl over 28 CV. SP column elution peak containing the target protein as confirmed *via* SDS-PAGE were concentrated to 1.0 ml and run on a Superdex200 (GE Healthcare) gel-filtration column in buffer A (plus 2 mM DTT for PKAc).

### Recombinant Ca_V_1.3 EFPreIQ protein expression and purification

The pET28HMT-EFPreIQ plasmid was transformed into *E. coli* Rosetta (DE3)pLysS competent cells and plated against chloramphenicol and kanamycin selection for 16 h at 37 °C. Colonies were then inoculated into a 100 ml 2 YT media (16 g/l tryptone, 10 g/l yeast extract, 5 g/l NaCl) starter culture and grown for 16 h at 37 °C. Ten microliters of starter culture was then inoculated into 1 L of 2 YT media and grown at 37 °C at 180 RPM until an *A*_600_ of 1.0 was reached. Cultures were then allowed to cool to 18 °C and induced to express with 0.4 mM IPTG for 16 h. The cells were then harvested and stored at −20 °C.

All steps in lysis and purification were performed at 4 °C or on ice. Upon thawing, cell pellets were lysed *via* sonication in a lysis buffer (20 mM Hepes [pH 7.4], 500 mM NaCl, 0.2% (v/v) EDTA-free protease inhibitor cocktail III (Millipore), 25 μg/ml DNaseI, and 50 μg/ml lysozyme). The lysed cells were pelleted *via* centrifugation (Beckman Coulter, Avanti J-E) at 40,000*g* for 35 min. The soluble lysate was filtered with a 0.45 μm nylon syringe filter, and then applied to HisTrap Fast Flow IMAC column (GE Healthcare) pre-equilibrated with buffer B (20 mM Hepes [pH 7.4], 500 mM NaCl). The column was washed with 15 CV of buffer B before eluting the recombinant protein using buffer B + 250 mM imidazole. The elution was pooled and applied to an Amylose Column (New England Biolabs) pre-equilibrated with buffer B + 15 mM β-ME. The column was then washed with 3 CV of buffer B before being eluted using buffer B + 15 mM β-ME and 20 mM maltose. The elution was then pooled, spiked with 2 mM EDTA, 5 mM DTT, and 2 mg of recombinant TEV protease before being dialyzed for 16 h against 20 mM Hepes [pH 7.4], 500 mM NaCl, and 10 mM β-ME. The dialyzed sample was then applied to a HisTrap Fast Flow IMAC column (GE Healthcare) pre-equilibrated with buffer B. The flowthrough containing the cleaved protein was collected, concentrated using an Amicon concentrator (10 K MWCO; Millipore), and applied to a preparative-grade Superdex75 column (GE Healthcare) pre-equilibrated with ITC/assay buffer. Monomeric EFPreIQ was then collected and concentrated using an Amicon concentrator (10 K MWCO; Millipore) to 1.5 mM for ITC experiments or to 800 μM for kinase assays.

### X-ray crystallography

The two major SP elution peaks for PKAc were used independently for crystallographic screening (Fig. S9). The two peaks likely correspond to two different species of PKAc: the first eluted species having more autophosphorylation sites than the second. Based on the electron densities we observed for ApoPKAc/AMP-PNP structures (peak 1) *versus* C2 (peak 2), we presume that the first peak corresponds to a species that is phosphorylated at residues Ser139, Thr197, and Ser338, while the second species is only phosphorylated at residues Thr197 and Ser338.

We obtained crystals in the presence of the Ca_V_1.2 Ser1981 peptide (RGFLRSASLGRRASFHL) using both forms of PKAc (Table S1). Peak 1 resulted in the formation of C1 crystal, and peak 2 formed crystal C2. All proteins used for crystallographic screening were concentrated and buffer exchanged with 20 mM bicine pH 8.0, 150 mM ammonium acetate, 4 mM TCEP. The PKAc samples were mixed with synthetic peptide either directly in the lyophilized powder or in a 5 mM stock solution in the same buffer. Final PKAc concentration for C1 crystal form was 300 μM with AMP-PNP, MgCl_2_, and Ser1981 peptide at molar ratio of 1:10:17:10. For the peptide cocrystal structure C2 final PKAc concentration was 250 μM in presence of AMP-PNP, MgCl2, and Ser1981 peptide for a molar ratio of 1:10:10:10. We also screened 300 μM PKAc (peak 1) in presence of a shorter Ser1718 peptide DIGPEIRRAISGDL, AMP-PNP, and MgCl_2_ at a molar ratio of 1:10:10:10. No peptide was found to be bound and this thus yielded the apoPKAc crystal form. 96-well plate low volume crystallization plates (Hampton Research) were all set up at room temperature using sitting drop method with ratios 1:1 and 1:2 for precipitant to protein, using a Phoenix crystallization robot (Art Robbins Instruments). All crystal plates were immediately stored at 4 °C. For all structures, the best diffracting crystals originated from the 1:2 precipitant to protein ratio and were transferred to a drop supplemented with 25% ethylene glycol as cryo-protectant.

Crystals were harvested and frozen in liquid nitrogen using Hampton or MiTeGen MicroMounts CryoLoops. All diffraction datasets were processed using HKL2000 (HKL Research Inc.). Best diffracting crystals for C1 appeared in condition 64 of ProComplex crystal screen (QIAGEN) with the following formulation: 0.1 M Hepes pH 7.0, and 18% (w/v) PEG 12,000 Da. Crystals for C2 appeared in condition 79 of JCSG + crystal screen (QIAGEN) with the following formulation: 0.1 M succinic acid pH 7.0, and 15% (w/v) PEG 3350 Da. The apoPKAc crystal was obtained in condition 64 of Classics crystal screen (QIAGEN) with the following formulation: 0.1 M Hepes pH 7.5, 10% (w/v) PEG 8000 Da. Datasets for C1 and apoPKAc were collected at Stanford Synchrotron Radiation Light source (beamlines 12-2 and BL9-2, respectively) at a wavelength of 0.979 Å, using a Dectris Pilatus3 6M detector, while data for the C2 crystal form were collected at the Advanced Photon Source (APS, 23 ID D) at a wavelength of 1.033 Å, also equipped with a Dectris Pilatus3 6M detector. All structures were solved *via* molecular replacement in Phaser (58), using PKAc derived from PDB 6MM5 as a search model. Restrained and translation, libration, and screw refinement was carried out using PHENIX (56, 57).

### ADP-GloMax kinase assays

Kinase assays were conducted using an ADP-Glo Kinase Assay Kit (Promega). The PKAc undergoes bisubstrate second order kinetics preferring the binding of ATP and Mg^2+^ to occur prior to substrate binding (59, 60, 61). Therefore, we performed assays with excess ATP (1 mM) and Mg^2+^ (5 mM) to saturate PKAc prior to substrate binding. Reagents were prepared as described in the kit, aliquoted, and frozen at −20 °C prior to use. Reactions were conducted in 384-well solid white polystyrene microplates (Corning). Peptide substrates and EFPreIQ were dissolved and/or diluted to 800 μM using 50 mM Hepes (pH 7.4), 150 mM KCl, 5 mM MgCl_2_, and 4 mM TCEP. Peptides containing tryptophan had their concentrations determined *via* absorbance at a wavelength of 280 nm using an extinction coefficient of 5500 M^−1^ cm^−1^. For peptides lacking tryptophan, the amount of volume needed to achieve a concentration of 800 μM was calculated prior to dissolving. The peptides/substrate was 2-fold serially diluted using the same buffer to create 11 reactions per curve ranging from 0 to 256 μM. ATP was added to each reaction to achieve a final concentration of 1 mM. Reactions were initiated by the addition of thawed PKAc at a final concentration of 3 nM at a final well volume of 20 μl. After allowing reactions to occur at room temperature for 30 min, 3 × 5 ml from each reaction in a curve was taken and mixed with 5 ml ADP-Glo Reagent for a total of three technical replicates per biological replicate. After 40 min, 10 ml of kinase detection reagent was added to each technical replicate and, after waiting 60 min, relative luminescence unit were measured using a VICTOR X4 Multilabel Plate Reader (PerkinElmer).

### Kinetic data acquisition and processing

The relative luminescence unit values were converted to specific activity values *via* interpolation of a standard curve that was created in accordance with the manufacturer’s protocol. The three technical replicates were averaged to calculate a biological replicate value and plotted against peptide concentration using GraphPad 9 (Prism) software (https://www.graphpad.com/updates/prism-900-release-notes). Technical replicate values resulting from experimental error were removed from the dataset and subsequent averaging. In some cases, all three technical replicates were removed resulting in only two biological replicates for a peptide concentration [Kemptide: 16 mM, Ser1718 peptide: 2 mM, EFPreIQ (Ser1535): 128 mM, Ser38 peptide: 2 mM, Ser300 peptide: 128 mM; 4 mM; and 1 mM] or only three biological replicates for the Ser272 peptide (128 mM). The Michaelis–Menten kinetics fitting function was used to generate a nonlinear regression to calculate both a K_m_ constant and V_max_ value for each biological replicate curve. These K_m_ constant and V_max_ values were averaged and SDs are reported. Figures displaying data points and Michaelis–Menten curves depict the average specific activity values calculated from all biological replicates in the three or four experiments (n = 3 or n = 4) per peptide (n = 4 for Ser1981 and Ser272 peptides, n = 3 for all others).

### Isothermal titration calorimetry

ITC experiments were conducted using a MicroCal iTC200 (GE Healthcare, now Malvern) instrument. The ITC/assay buffer was used to dissolve peptides to a concentration of 1.25 to 4.3 mM. Substrate concentrations were optimized and chosen based on how much was needed to achieve sufficient signal to noise ratio. Rad Ser25 peptide contained a tryptophan residue, which allowed us to determine its concentration *via* 280 nm absorbance readings using an extinction coefficient of 5500 M^−1^ cm^−1^. For peptides lacking a tryptophan or tyrosine residue (Ser1981 WT and mutant peptides, Ser1718 peptide, Ser272 peptide, and Ser300 peptide), the amount of volume needed to achieve the desired concentration was calculated prior to diluting. Peptides were received as powder in 1 mg aliquots and using molecular weights of the peptide, the number of moles in each aliquot was determined. The number of moles was used to determine how much volume was required to achieve the desired concentration. AMP-PNP (Millipore Sigma) was dissolved in ITC/Assay buffer to a concentration of 100 mM, aliquoted in 20 μl volumes, and stored at −70 °C. Prior to each run, both titrant and titrand solutions were supplemented with AMP-PNP to a final concentration of 500 μM. The titration experiments were conducted at 4 °C with a stirring speed of 500 RPM. Each titration experiment was comprised of 20 injections with 230 s in between each injection. Each injection totalled 2.0 μl over 4 s except the first injection being 0.4 μl over 0.8 s. Background runs of peptide/substrate into ITC/assay buffer were conducted to generate a linear regression using the isotherm to subtract background heats from experimental runs. Peptides displaying large background heats were dialyzed in ITC/assay buffer for 60 min to remove contaminants remaining from peptide synthesis. The peptide into PKAc runs (experimental runs) utilized the same settings. The data was processed using Origin (Version 7.0, OriginLab) software (https://www.originlab.com/origin). For runs utilizing peptides that did not provide a significant absorbance at 280 nm, the concentrations were manually adjusted to achieve a stoichiometry of N = 1 (Rad Ser272 and Ca_V_1.2 Ser1981 peptides).

## Data availability

The atomic coordinates and structure factors for the PKAc in complex with CaV1.2 S1981 peptide C1 and C2, and the ApoPKA/AMP-PNP structure have been deposited in the Protein Data Bank with accession codes PDB: 8UKP, 8UKO, and 8UKN, respectively (http://www.rcsb.org/).

## Supporting information

This article contains supporting information.

## Conflict of interest

The authors declare that they have no conflicts of interest with the contents of this article.
