# Supplementary material for: Crystallographic, kinetic, and calorimetric investigation of PKA interactions with L-type calcium channels and Rad GTPase
Source: J Biol Chem. 2024 Nov 29;301(1):108039. doi: 10.1016/j.jbc.2024.108039 (PMC11728977; doi:10.1016/j.jbc.2024.108039)
Supplement: Supplemental information [file mmc1.docx]

**Supporting Information**

***Human CaV1.2 CTD (Q13936-1)***  DYLTRDW**S**ILGPHHLDEFKRIWAEYDPEAKGRIKHLDVVTLLRRIQPPLGFGKLCPHRVA **1587**

***Rabbit CaV1.2 CTD (P15381-1)*** DYLTRDW**S**ILGPHHLDEFKRIWAEYDPEAKGRIKHLDVVTLLRRIQPPLGFGKLCPHRVA **1569**

***Human CaV1.3 CTD (Q01668-1)***  DYLTRDW**S**ILGPHHLDEFKRIWSEYDPEAKGRIKHLDVVTLLRRIQPPLGFGKLCPHRVA **1527**

**********************:*************************************

***Human CaV1.2 CTD (Q13936-1)***  CKRLVSMNMPLNSDGTVMFNATLFALVRTALRIKTEGNLEQANEELRAIIKKIWKRTSMK **1647**

***Rabbit CaV1.2 CTD (P15381-1)*** CKRLVSMNMPLNSDGTVMFNATLFALVRTALRIKTEGNLEQANEELRAIIKKIWKRTSMK **1629**

***Human CaV1.3 CTD (Q01668-1)***  CKRLVAMNMPLNSDGTVMFNATLFALVRTALKIKTEGNLEQANEELRAVIKKIWKKTSMK **1587**

*****:*************************:****************:******:****

***Human CaV1.2 CTD (Q13936-1)***  LLDQVVPPAGDDEVTVGKFYATFLIQEYFRKFKKRKEQGLVGK-PSQRNALSLQAGLRTL **1706**

***Rabbit CaV1.2 CTD (P15381-1)*** LLDQVVPPAGDDEVTVGKFYATFLIQEYFRKFKKRKEQGLVGK-PSQRNALSLQAGLRTL **1688**

***Human CaV1.3 CTD (Q01668-1)***  LLDQVVPPAGDDEVTVGKFYATFLIQDYFRKFKKRKEQGLVGKYPAKNTTIALQAGLRTL **1647**

**************************:**************** :..:::********

***Human CaV1.2 CTD (Q13936-1)***  HDIGPEIRRAI**S**GDLTAEEELDKAMKEAVSAASEDDIFRRAGGLFGNHVSYYQSDGRSAF **1766**

***Rabbit CaV1.2 CTD (P15381-1)*** HDIGPEIRRAI**S**GDLTAEEELDKAMKEAVSAASEDDIFRRAGGLFGNHVSYYQSDSRSAF **1748**

***Human CaV1.3 CTD (Q01668-1)***  HDIGPEIRRAI**S**CDLQDDEP------EETKREEEDDVFKRNGALLGNHVNHVNSDRRDSL **1701**

************ ** :* .. .***:*:* *.*:****.: :** *.::

***Human CaV1.2 CTD (Q13936-1)***  PQTFTTQRPLHINKAGSSQG-DTESPSHEKLVDSTFTPSSYS-------STGSNANINNA **1818**

***Rabbit CaV1.2 CTD (P15381-1)*** PQTFTTQRPLHISKAGNNQG-DTESPSHEKLVDSTFTPSSYS-------STGSNANINNA **1800**

***Human CaV1.3 CTD (Q01668-1)***  QQTNTTHRPLHVQRPSIPPASDTEKPLFPPAGNSVCHNHHNHNSIGKQVPTSTNANLNNA **1761**

** **:****:.: . . ***.* . :*: *.:***:***

***Human CaV1.2 CTD (Q13936-1)***  NNTA--LGRLPRPAGYPSTVSTVEGHGPPLSPAIRVQEVAWKLSSNRERHVPMCEDLELR **1876**

***Rabbit CaV1.2 CTD (P15381-1)*** NNTA--LGRLPRPAGYPSTVSTVEGHGSPLSPAVRAQEAAWKLSS--------------- **1843**

***Human CaV1.3 CTD (Q01668-1)***  NMSKAAHGKRPSIGNL-EHVS-ENGHHSSHKHD---REPQRRSSVKRTR----YYETYIR **1812**

* : *: * .. . ** :** . :* : *

***Human CaV1.2 CTD (Q13936-1)***  RDSGSAGTQAHCLLLRK----ANPSRCHSRESQAAMAGQEETSQDETYEVKMNHDTEACS **1932**

***Rabbit CaV1.2 CTD (P15381-1)*** ------------------------KRCHSQESQIAMACQEGASQDDNYDVRIGEDAECCS **1879**

***Human CaV1.3 CTD (Q01668-1)***  SDSGDEQLPTICREDPEIHGYFRDPHCLG--------EQEYFSSEECYEDD--------S **1856**

:* . ** *.:: *: *

***Human CaV1.2 CTD (Q13936-1)***  EPSLLSTEMLSYQDDENR--------QLTLP----EED----KRDIRQSPKRGFLR-SAS **1975**

***Rabbit CaV1.2 CTD (P15381-1)*** EPSLLSTEMLSYQDDENR--------QLAPP----EEE----KRDIRLSPKKGFLR-SAS **1922**

***Human CaV1.3 CTD (Q01668-1)***  SPTWSRQNYGYYSRYPGRNIDSERPRGYHHPQGFLEDDDSPVCYDSRRSPRRRLLPPTPA **1916**

.*: : *. .* * *:: : * * **:: :* : :

***Human CaV1.2 CTD (Q13936-1)***  LGRRA**S**FHLECLKRQKDRGG-----DISQKTVLPLHLVHHQALAVAGLSPLLQRSHSPAS **2030**

***Rabbit CaV1.2 CTD (P15381-1)*** LGRRA**S**FHLECLKRQKNQGG-----DISQKTVLPLHLVHHQALAVAGLSPLLQRSHSPTS **1977**

***Human CaV1.3 CTD (Q01668-1)***  SHRRS**S**FNFECLRRQSSQEEVPSSPIFPHRTALPLHLMQQQIMAVAGLDSSKAQKYSPSH **1976**

**:**::***:**..: : ::*.*****:::* :*****. :.:**:

***Human CaV1.2 CTD (Q13936-1)***  FPRPFATPPATPGSRGWPPQPVPTLRLEGVESSEKLNSSFPSIHCGSWA-ETTPGGGGSS **2089**

***Rabbit CaV1.2 CTD (P15381-1)*** LPRPCATPPATPGSRGWPPQPIPTLRLEGADSSEKLNSSFPSIHCGSWSGENSPCRGDSS **2037**

***Human CaV1.3 CTD (Q01668-1)***  STRSWATPPATPPYRDWTPCYTPLIQVEQSEALDQVNGSLPSLHRSSWYTDE-PD----I **2031**

* ******* *.* * * :::* :: :::*.*:**:* .** : *

***Human CaV1.2 CTD (Q13936-1)***  AARRVRPVSLMVPSQAGAPGRQFHGSASSLVEAVLISEGLGQFAQDPKFIEVTTQELADA **2149**

***Rabbit CaV1.2 CTD (P15381-1)*** AARRARPVSLTVPSQAGAQGRQFHGSASSLVEAVLISEGLGQFAQDPKFIEVTTQELADA **2097**

***Human CaV1.3 CTD (Q01668-1)***  SYRTFTPASLTVPSSFRNKNSDKQRSADSLVEAVLISEGLGRYARDPKFVSATKHEIADA **2091**

: * *.** ***. . : : **.*************::*:****:..*.:*:***

***Human CaV1.2 CTD (Q13936-1)***  CDMTIEEMESAADNILSGGAPQSPNGALLPFVNCRDAGQDRAGGEEDA---GCVRARGRP **2206**

***Rabbit CaV1.2 CTD (P15381-1)*** CDLTIEEMENAADDILSGGARQSPNGTLLPFVNRRDPGRDRAGQNEQDASGACAPGCGQ- **2156**

***Human CaV1.3 CTD (Q01668-1)***  CDLTIDEMESAASTLLNGNVRPRANGDVGPLSHRQDYELQDFGPGYSDE----EPDPGR- **2146**

**:**:***.**. :*.*.. ** : *: : :* : * . *:

***Human CaV1.2 CTD (Q13936-1)***  SEEELQDSRVYVSSL **2221**

***Rabbit CaV1.2 CTD (P15381-1)*** SEEALADRRAGVSSL **2171**

***Human CaV1.3 CTD (Q01668-1)***  DEEDLADEMICITTL **2161**

.** * * :::*

**Figure S1. Sequence alignment for the CTD regions of Ca_V_1.2 and Ca_V_1.3.** Sequence alignment for the C-terminal domain (CTD) regions of Human (uniprot: Q13936-1), Rabbit (uniprot: P15381-1) Ca_V_1.2 as well as the human (uniprot: Q01668-1) Ca_V_1.3 sequence. All three phosphorylation sites (S1535, S1718, and S1981) discussed here for Ca_V_1.2 are indicated with red bold font and underlined.

**
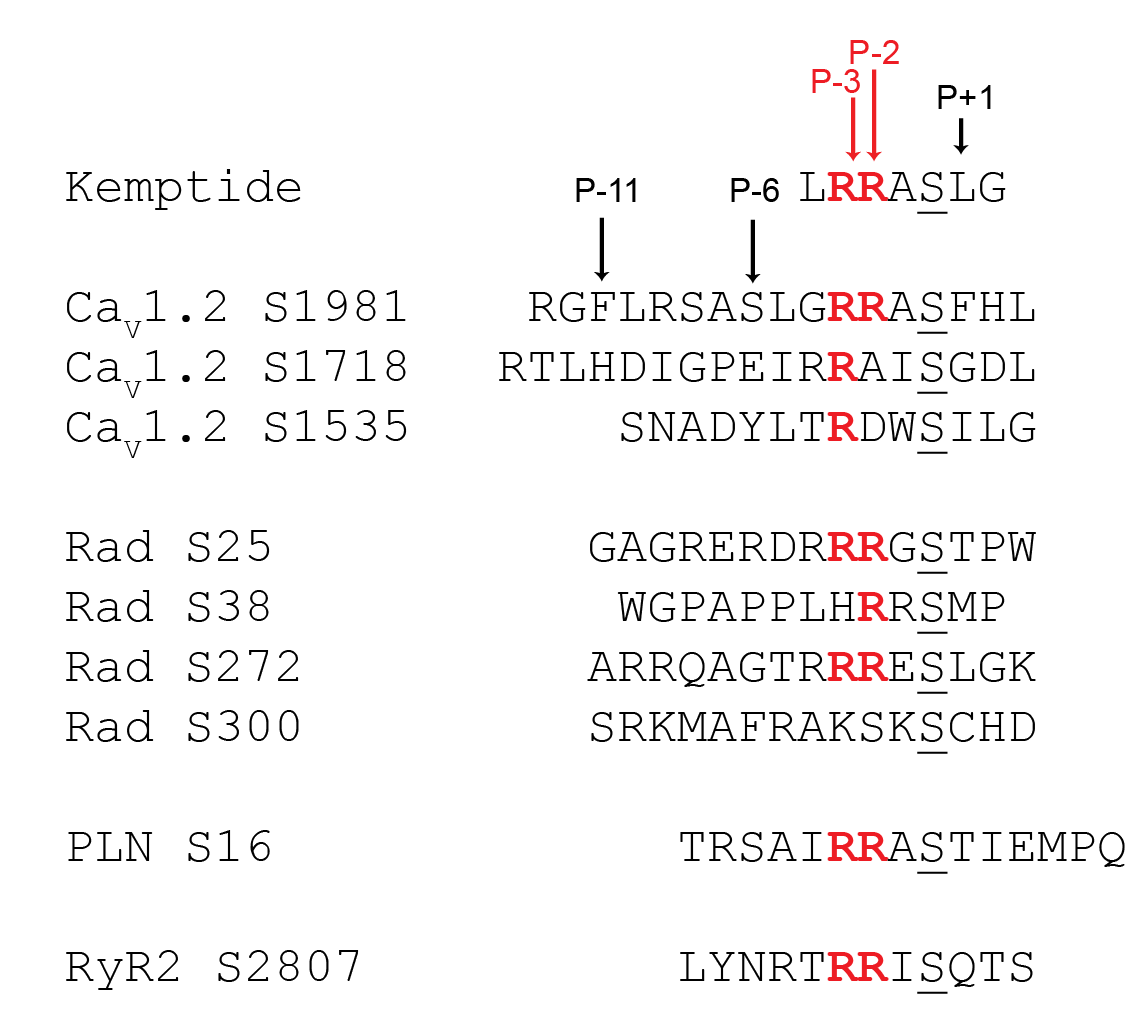
**

**Figure S2. Sequence alignment of peptides used in this study.** The target serine is underlined, and Arg residues in the P-2 and P-3 sites are highlighted in red. For the Ser1535 site in Ca_V_1.2, the downstream Ca_V_1.3 EF-hand domain is not shown. The first three residues ‘SNA’ are a leftover from the vector used to express the protein (see Methods). Further residues upstream in this peptide are part of the transmembrane region and were therefore not used, as they would cause issues with solubility and would not normally be accessible for binding PKAc.


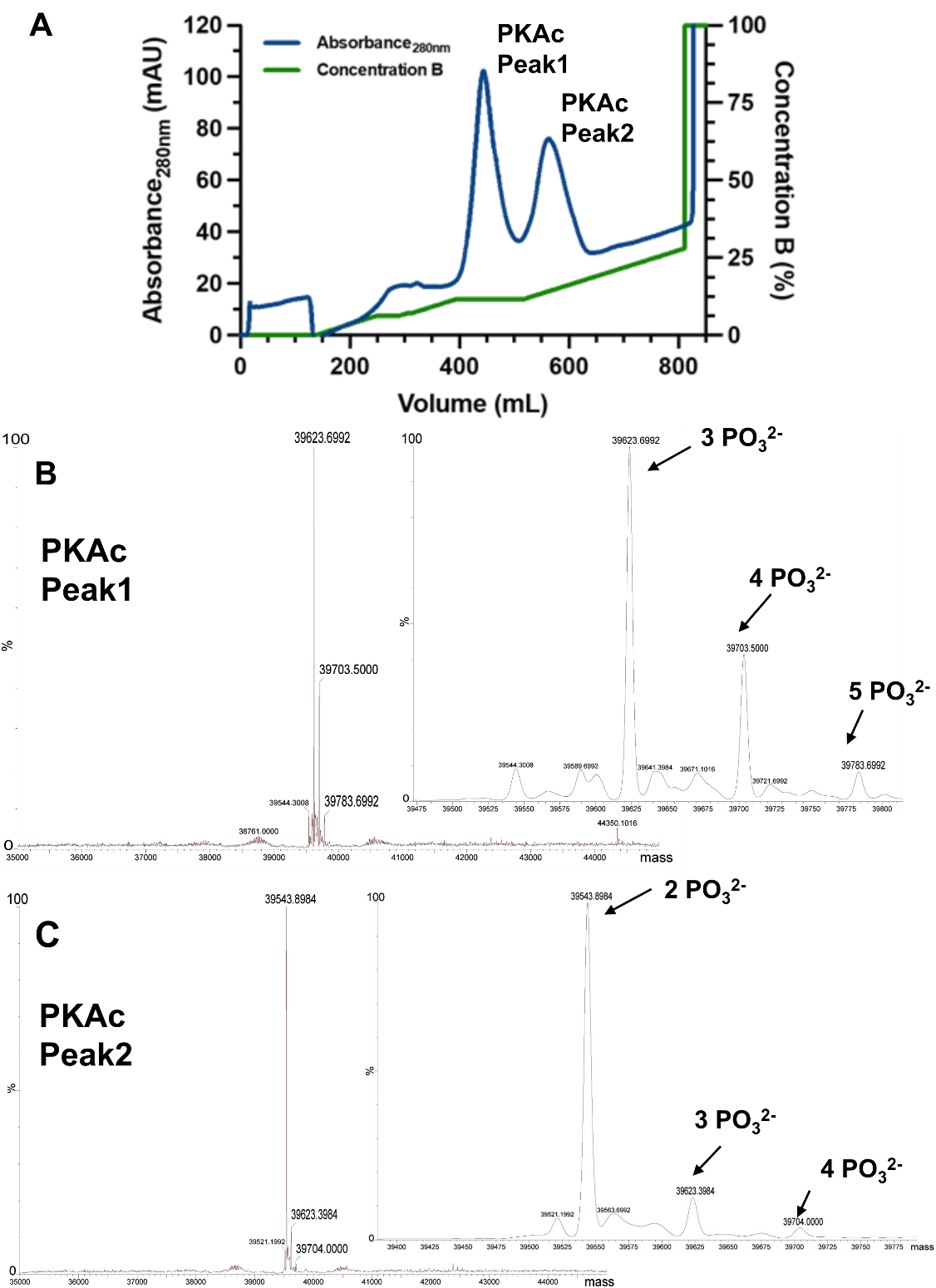


**Figure S3. Cationic Exchange Chromatograph of PKAc. A,** representative chromatogram of the chromatography step is shown with the UV_280_ (mAU) shown in blue (left y-axis) and percent concentration of buffer B (15 mM potassium-phosphate buffer [pH 6.3], 1 M KCl, and 10 mM βME) applied to the column shown in green (right y-axis). Two predominant species elute closely together, requiring the holding of the salt gradient to ensure sufficient separation of the two species.

***B, C,*** Mass spectrometry analysis of the two main peaks shown in panel A. The insets show a zoomed-in view of the main peaks. Although not quantitative, these suggest that peak 1 and peak 2 mostly contains tripe-phosphorylated and double-phosphorylated PKAc, in agreement with electron densities observed in the corresponding crystal structures. These spectra were collected on a Waters Xevo G2S QTof instrument equipped with a nanoAquity HPLC.

**Figure S4. Structures with the highest structural similarity to ApoPKAc2 molecule (PDB:** **8UKN, Chain F).** The top closest structural matches of ApoPKAc2 RMSD values 1.0 Å or lower overlaid on the ApoPKAc2 structure. The top apo and binary (inhibitor peptide-bound, nucleotide-unbound) structures are shown on the left and the top ternary structures (complexes including AMP-PNP and RyR2 substrate) on the right.


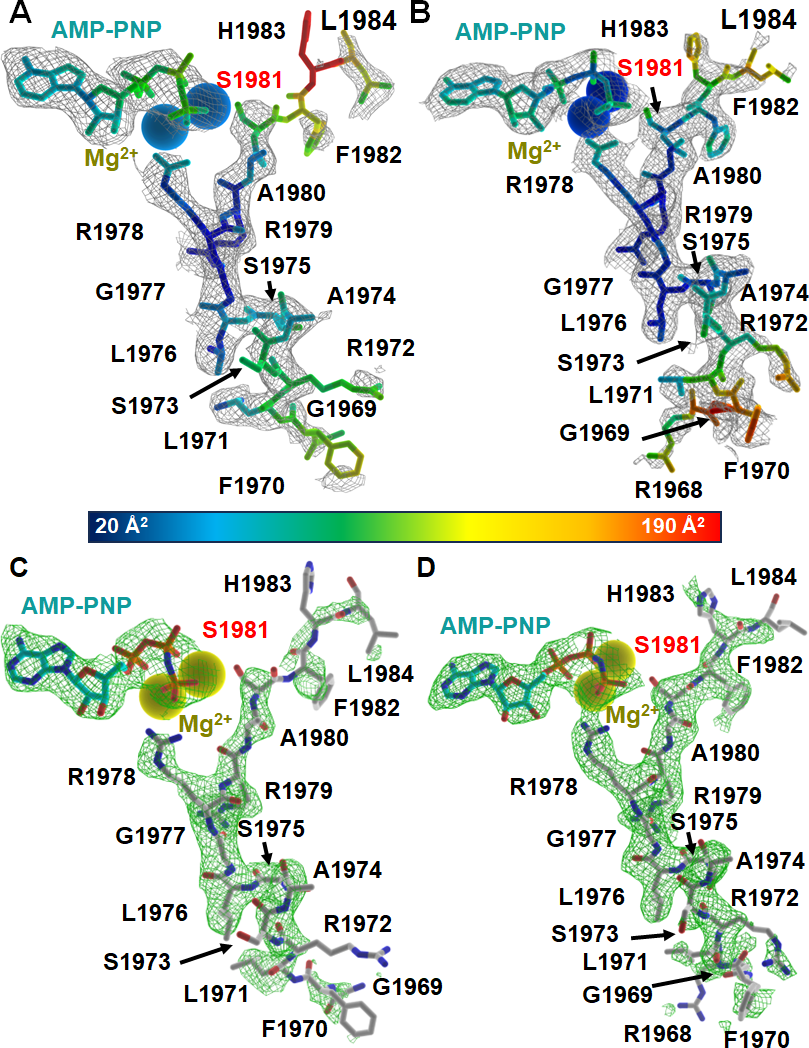


**Figure S5. Map quality. A** and **B**, 2Fo-Fc density (contoured at 0.7 σ) and B-factor coloring for the Ca_V_1.2 S1981 peptide, Adenylyl-imidodiphosphate (AMP-PNP), and the two magnesium (Mg^2+^, spheres) ions in Complex1 (A) and Complex2 (B). The color scale for the B-factors is shown at the bottom. CaV1.2 amino acids shown in black while the S1981 PKA target site labelled in red. **C** and **D** Corresponding omit Fo-Fc density map (contoured at 2 σ) for the same complexes shown in panels A and B, respectively. Colour scheme: Ca_V_1.1 carbons: Grey, AMP-PNP carbons: Cyan, Nitrogen: Blue, Oxygen: Red, Phosphorous: Orange, Magnesium (Mg^2+^): yellow spheres.

**
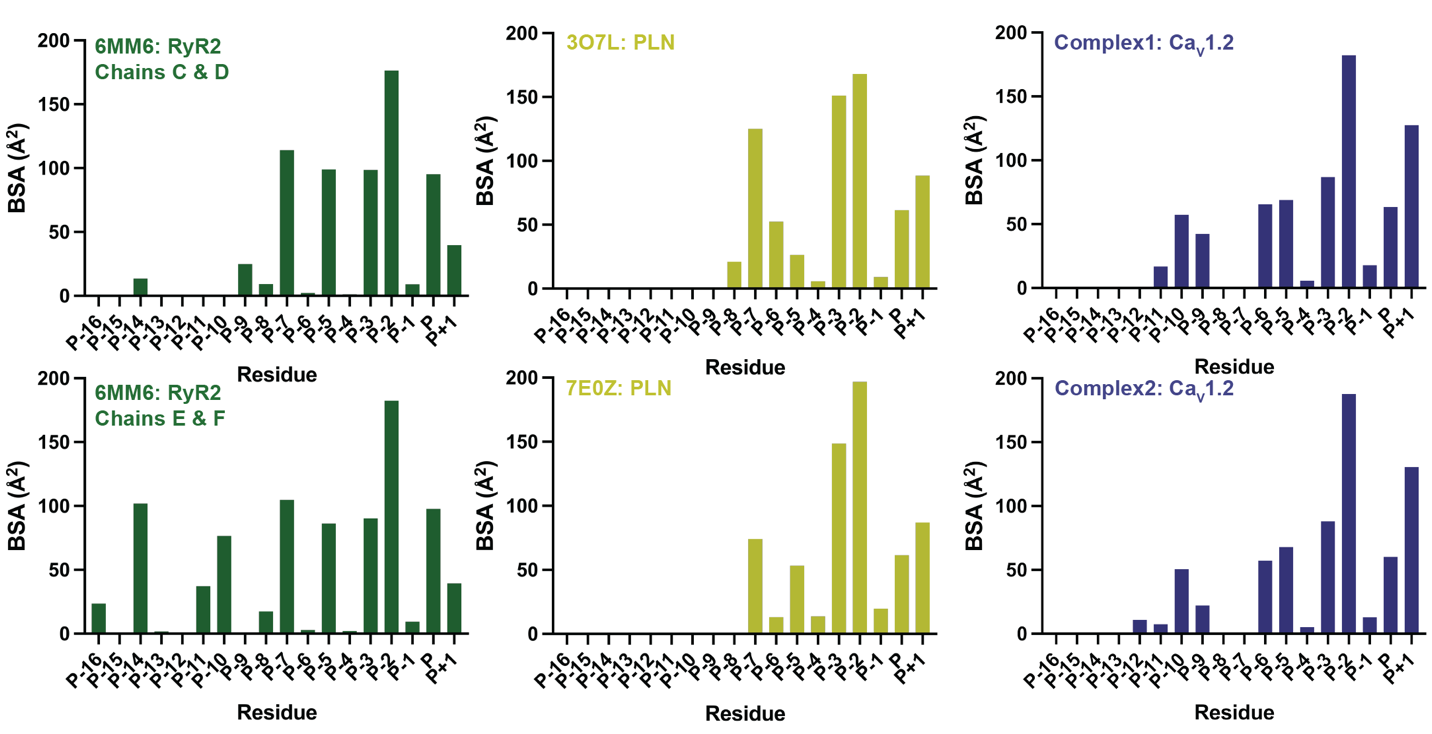
**

**Figure S6. Buried surface area plots of PKAc:substrate complexes.** Graphs depict how much each residue in each substrate is buried by PKAc.

**Figure S7. 2D LIGPLOT diagram of interactions between PKAc and S1981 peptide.**

Blue text represents CaV1.2 peptide residues. Black and green text correspond to residues from the PKA catalytic domain. Green residues are specifically involved in hydrogen bonds and salt bridge interactions with the CaV1.2 peptide, while black residues participate in hydrophobic contacts with CaV1.2. Hydrogen bonds and coordination to the Mg^2+^ ion are shown as green lines, while the spoked arcs represent protein residues making nonbonded contacts with the ligand. Red lines represent salt-bridge interactions between PKA residues and CaV1.2.


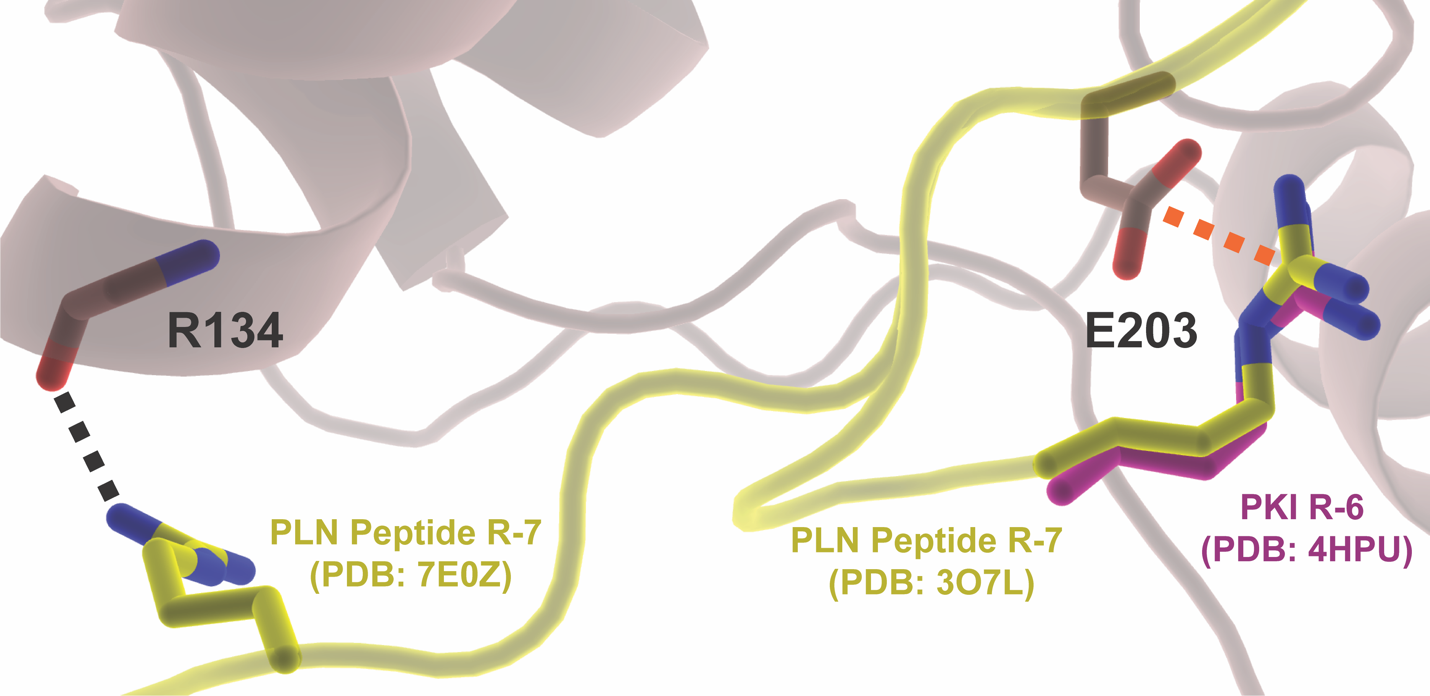


**Figure S8. Differences between R-7 residues of PKAc:PLN ternary structures.** The positions of the R-7 side chains in both PKAc:PLN structures are shown. The R-7 of one PLN structure (PDB: 3O7L) is positioned identically to R-6 of PKI (PDB: 4HPU). The hydrogen bond observed in the first PLN structure is depicted as a dotted black line and the salt bridge in the second structure as an orange dashed line drawn from one charge centre to the other.


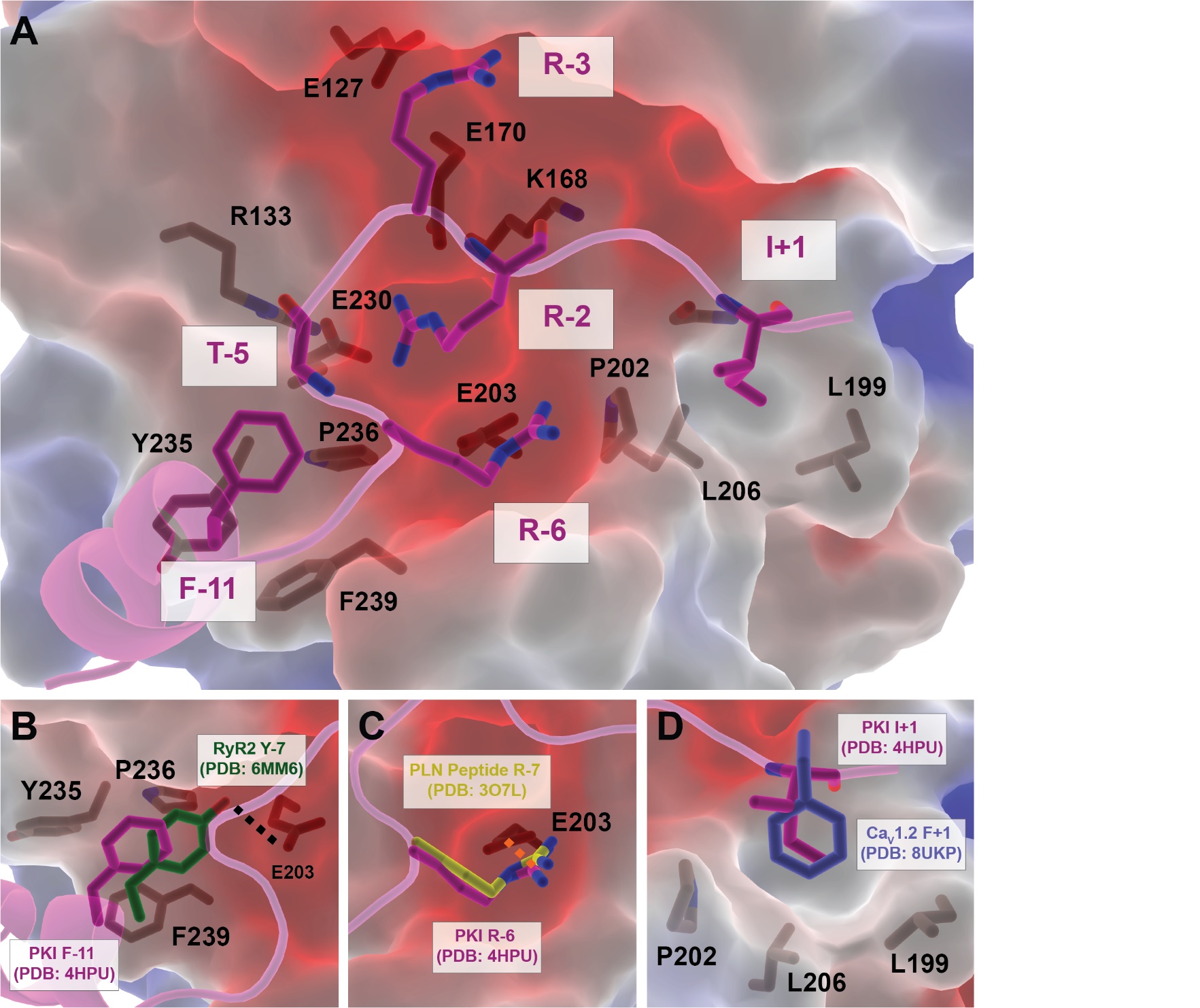


**Figure S9. Interactions of PKI peptide with PKAc active site in comparison with PKAc: substrate complexes. *A,*** PDB: 4HPU structure depicts PKAc in complex with substrate competent-PKI. PKAc is coloured according to electrostatic potential. PKI is depicted in cartoon representation. PKAc and PKI residues involved in interactions are shown as sticks. ***B****,* F-11 of PKI slots into a shallow hydrophobic groove in the PKAc active site formed by Y235, P236, and F239. Y-7 of RyR2 (PDB: 6MM6) engages similarly with an additional hydrogen bond (black dotted line) to E203. ***C****,* The R-7 in one PKAc:PLN ternary structure (PDB: 3O7L) is positioned nearly identically to R-6 of PKI and forms a salt bridge (orange dotted line) with E203. ***D****,* A hydrophobic pocket formed by P202, L206, and L199 of PKAc is occupied by I+1 in PKI and F+1 in Ca_V_1.2 (PDB: 8UKP).

**Table S1. Crystallographic Table.**

| **Crystal** | **PKAc-S1981**  **Complex1** | **PKAc-S1981**  **Complex2** | **Apo/AMP-PNP PKAc** |
| --- | --- | --- | --- |
| PDB codes | 8UKP | 8UKO | 8UKN |
| Resolution (Å) | 35.0–2.85 (2.92–2.85)[^a^](https://www.sciencedirect.com/science/article/pii/S1097276519303119#tblfn1) | 35.0–2.99 (3.06–2.89)[^a^](https://www.sciencedirect.com/science/article/pii/S1097276519303119#tblfn1) | 35.0–2.75 (2.81–2.75)[^a^](https://www.sciencedirect.com/science/article/pii/S1097276519303119#tblfn1) |
| Space group | P 4_1_2_1_2 | *I* 4_1_ | P2_1_ |
| a (Å) | 118.63 | 118.058 | 61.8 |
| b (Å) | 118.63 | 118.058 | 139.8 |
| c (Å) | 57.11 | 58.0 | 107.4 |
| α, β, γ (°) | 90.0, 90.0, 90.0 | 90.0, 90.0, 90.0 | 90.0, 102.8, 90.0 |
| Volume Å^3^ | 8.05 × 10^5^ | 8.08 × 10^5^ | 9.05 × 10^5^ |
| Wavelength (Å) | 0.979 | 0.979 | 1.033 |
| No. of molecules/A.U. | 1 | 1 | 4 |
| Unique reflections | 9,956 | 9,121 | 44,968 |
| Redundancy | 13.6 (14.1) | 4.7 (4.6) | 3.7 (3.3) |
| 〈I/σ(I)〉 | 35.4 (1.37) | 25.2 (1.4) | 16.1 (2.15) |
| CC1/2 (%)[^b^](https://www.sciencedirect.com/science/article/pii/S1097276519303119#tblfn2) | 99.7 (57.1) | 99.5 (34.2) | 99.7 (83.0) |
| Rpim (%)[^c^](https://www.sciencedirect.com/science/article/pii/S1097276519303119#tblfn3) | 2.80 (100.9) | 3.00 (58.3) | 4.50 (29.7) |
| Completeness (%) | 100.0 (100.0) | 99.9 (100.0) | 97.2 (85.1) |
| Protein atoms | 2,678 | 2,781 | 10,587 |
| Water oxygen atoms | 12 | 8 | 136 |
| Ligands or ions | 33 | 33 | 35 |
| Ramachandran outliers | 0 | 0 | 0 |
| Solvent content (%)[^d^](https://www.sciencedirect.com/science/article/pii/S1097276519303119#tblfn4) | 53.1 | 51.5 | 58.8 |
| Wilson *B*-factor (Å^2^) | 52.8 | 50.9 | 42.7 |
| ***Refinement*** |  |  |  |
| R_work_ (%) | 24.0 | 21.0 | 19.5 |
| R_free_ (%)[^e^](https://www.sciencedirect.com/science/article/pii/S1097276519303119#tblfn5) | 28.1 | 25.9 | 23.2 |
| RMSD bond lengths (Å) | 0.002 | 0.002 | 0.004 |
| RMSD bond angles (°) | 0.48 | 0.43 | 0.63 |
| Mean B-factor (Å^2^) | 65.0 | 64.0 | 47.0 |
| Protein atoms (Å^2^) | 65.7 | 64.7 | 47.1 |
| Water atoms (Å^2^) | 33.8 | 28.5 | 35.8 |
| Ligands and ions (Å^2^) | 70.9 | 41.5 | 91.7 |

*Two protein kinase A catalytic domain (PKAc) crystal structures were solved in complex with a peptide containing the CaV1.2 S1981 target site. These are referred to as crystal form 1 and 2 (CF1 and CF2). The peptide used was RGFLRSASLGRRASFHL (1968-1984) respectively. RMSD, root mean square deviation. NA: Not applicable.*

*^a^ Values in the parentheses refer to the highest resolution shell*

*^b^ CC_1/2_ = σ_i_ ^2^ / (σ_i_ ^2^ + σ_j_ ^2^), where σ_j_ denotes mean error for half-dataset* ^3^

*^c^ Rpim = ∑_hkl_ (1/n − 1)^1/2^ ∑_i_ |I_hkl, i_ − [I_hkl_]| / ∑_hkl_ ∑_i_ I_hkl, I_, where [I_hkl_] is the average of Friedel-related observations (i) of a unique reflection (hkl)*

*^d^ Estimated using sfcheck* ^4^

*^e^ 5% of reflections were omitted for R_free_ calculations*

**Table S2. Distances Between Residues Used to Determine Open/Intermediate/Closed States**

| Crystal Structure | Molecule Name (Chain) | G52-D166 (Cα-Cα) | S53-G186 (Cα-Cα) | H87−T197 (Nε2-PO4) | E170−Y330 (C=O−OH) | State |
| --- | --- | --- | --- | --- | --- | --- |
| Apo/AMP-PNP PKAc  (PDB: 8UKN) | ApoPKAc 1 (C) | 19.5 | 14.1 | 7.3 | n/a | Open |
|  | ApoPKAc 2 (F) | 19.1 | 14.0 | 6.6 | n/a | Open |
|  | ApoPKAc 3 (H) | 19.6 | 14.2 | 8.0 | n/a | Open |
|  | AMP-PNP PKAc (D) | 17.4 | 13.1 | 6.6 | n/a | Intermediate |
| PKAc-S1981  Complex1  (PDB: 8UKP) | Complex 1 (E) | 16.1 | 12.9 | 3.1 | n/a | Intermediate |
| PKAc-S1981  Complex2 (PDB: 8UKO) | Complex 2 (E) | 16.5 | 13.5 | 5.5 | 9.1 | Intermediate |

**Table S3. Interactions Between the Ca_V_1.2 S1981 Peptide and PKAc**

| Ca_V_1.2 S1981 Peptide Residue | Interaction Type | Complex 1 PKAc Residues (PDB: 8UKP) | Complex 2 (PDB: 8UKO) |
| --- | --- | --- | --- |
| G1969 | VDW | F239 | - |
| F1970 | VDW | F239 | F239 |
| L1971 | VDW | Y235, F239 | Y235, F239 |
| R1972 | SB | - | D241 |
|  | HB^NH1^ | - | D241^OD2^ |
|  | VDW | D241 | D241 |
| S1975 | VDW | R133, E203, P236, F239 | R133, E203, P236, F239, A240 |
| L1976 | HB^O^ | R133^NE^ | R133^NE^ |
|  | VDW | F129, R133, G234, Y235, P236, | F129, R133, G234, Y235, P236 |
| G1977 | VDW | F129, E170 | F129, E170, R133 |
| R1978 | SB | E127 | E127 |
|  | HB^NE^ | E127^OE2^ | E127^OE1^ |
|  | HB^NH2^ | E127^OE2^ | E127^OE2^ |
|  | VDW | E127, F129, E170, N171 | E127, F129, E170 |
| R1979 | SB | E170, E230 | E170, E203, E230 |
|  | HB^N^ | E170^OE2^ | E170^OE2^ |
|  | HB^NE^ | E170^OE2^ | E170^OE2^ |
|  | HB^NH1^ | - | E203^OE1^ |
|  |  | - | E230^OE1^ |
|  |  | E230^OE2^ | - |
|  | HB^NH2^ | E170^OE1^ | E170^OE2^ |
|  |  | E230^OE2^ | E230^OE2^ |
|  | HB^O^ | K168^NZ^ | K168^NZ^ |
|  | VDW | F129, R133, K168, P169, E170, T201, E203, Y204, E230, P236 | F129, R133, K168, P169, E170, T201, E203, Y204, E230, P236 |
| A1980 | VDW | K168, G200, T201 | K168, G200, T201, P202 |
| S1981 | VDW | D166, K168, F187, G200, T201 | D166, K168, F187, G200, T201 |
| F1982 | HB^N^ | G200^O^ | G200^O^ |
|  | HB^O^ | G200^N^ | - |
|  | VDW | F187, L198, C199, G200, T201, P202, L205, Y247 | F187, L198, C199, G200, T201, P202, L205, Y247 |

Hydrogen bonds and salt bridge interactions were determined using PDBePISA^10^. Van der Waals (VDW) Interactions were determined using CONTACT^11^. Hydrogen bonding donor and acceptor atoms are listed for hydrogen bonds as superscripts. The hydrogen bond (HB) distance cut-off was set to 3.5 Å and the salt bridge (SB) distance cut-off (measured from points at each residue’s charge centre) was set to 4.0 Å. Interactions unique to each complex are listed in red.

**Table S4. Human Ca_V_1.2 sequence variants of PKAc binding region.**

| Mutation | Disease | Source | Predicted Effect |
| --- | --- | --- | --- |
| G1969A | Long QT syndrome | Clinvar/gnomAD | Affects vdW contacts with F239 of PKAc |
| R1972H | Long QT syndrome | Clinvar/gnomAD | Ablates vdW contacts with D241 side chain of PKAc |
| R1972C | Timothy syndrome, Cardiovascular phenotype (not provided) Long QT syndrome, Arrhythmogenic right ventricular cardiomyopathy, History of neurodevelopmental disorder (not specified) | Clinvar/gnomAD | Ablates vdW contacts with D241 of PKAc |
| A1974P | Cardiovascular phenotype (not provided), Long QT syndrome, Timothy syndrome, Brugada syndrome | Clinvar/gnomAD | Affects the positioning of Ca_V_1.2 by restricting backbone movement, and perturbing binding. |
| G1977S | Timothy syndrome, Long QT syndrome, Brugada syndrome | Clinvar | Introduce steric impediments, abrogating vdw contacts with F129 and E170. |
| G1977D | Long QT syndrome | Clinvar/gnomAD | Introduce steric impediments, abrogating vdw contacts with F129 and E170. |
| R1978Q | Timothy syndrome, Brugada syndrome,  Cardiovascular phenotype, Long QT syndrome | Clinvar/gnomAD | Affects salt bridging interaction of R1978 with electronegative pocket of PKAc (E170, E203, E230). |
| R1979K | Long QT syndrome | Clinvar | Affects salt bridge formed with E127 of PKAc. |
| S1981P | Long QT syndrome | Clinvar | Phosphorylation-incompetent. |
| S1981F | Long QT syndrome | Clinvar/gnomAD | Phosphorylation-incompetent. |

Sequence variants in the Ca_V_1.2 Ser1981 peptide according to the Clinvar and gnomAD databases, showing the disease description and prediction of the impact of the variants based on the structure presented in this manuscript.
